# Supplementary material for: Interaction of carbamoyl-phosphate synthase 1 with agmatinase in the liver of torpid bats
Source: Curr Zool. 2026 Jan 6;72(5):703–13. doi: 10.1093/cz/zoag002 (PMC13690593; doi:10.1093/cz/zoag002)
Supplement: Supplementary file 1 [file zoag002_supplementary_data.zip › Supplementary Information 1.pdf]

Myotis ricketti

All WB images

CPS1

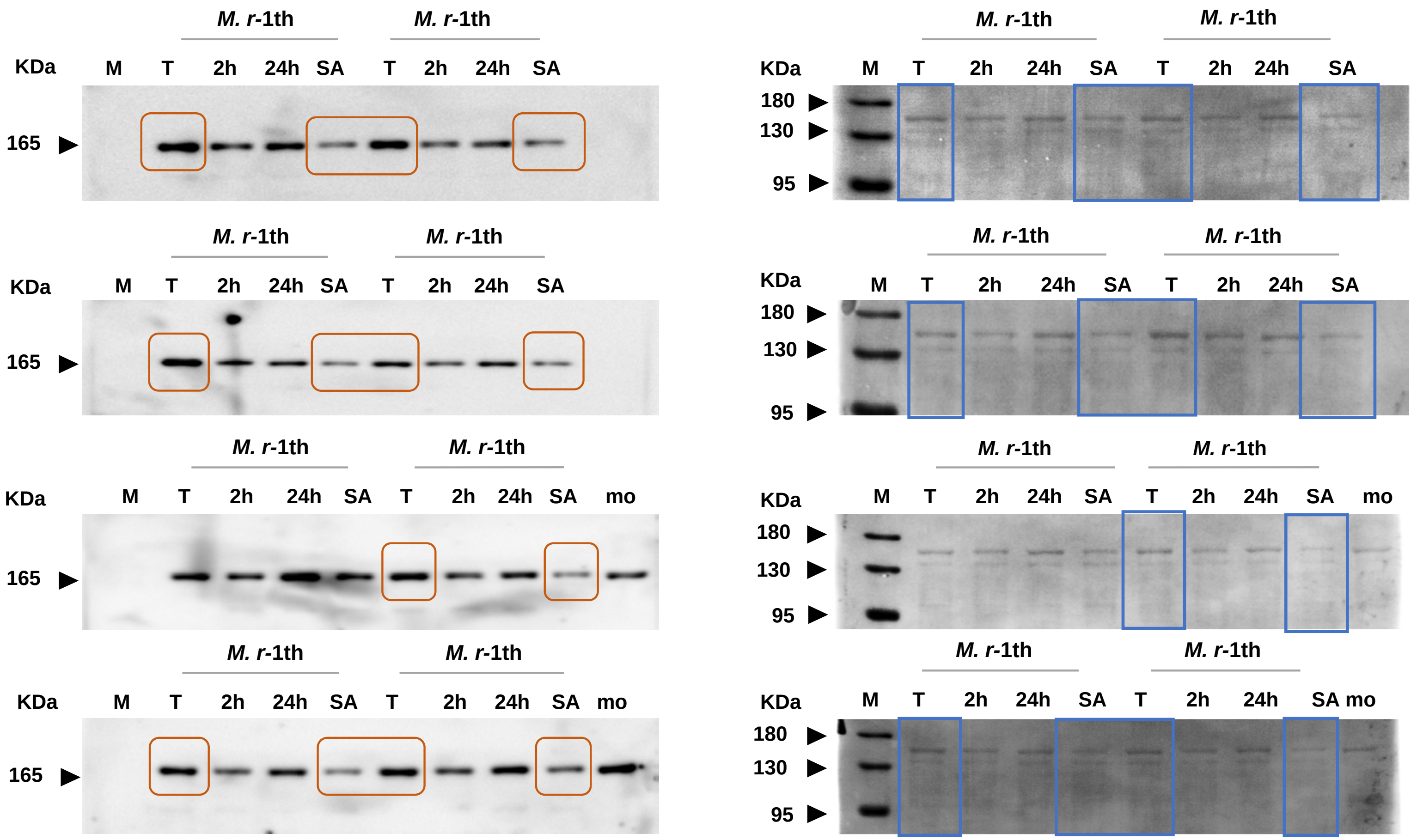

*Different numbers represent different bat individuals.
*M: marker, T: torpor, 2h: 2 hours after arousal, 24h: 24 hours after arousal; SA: summer active state

CPS1

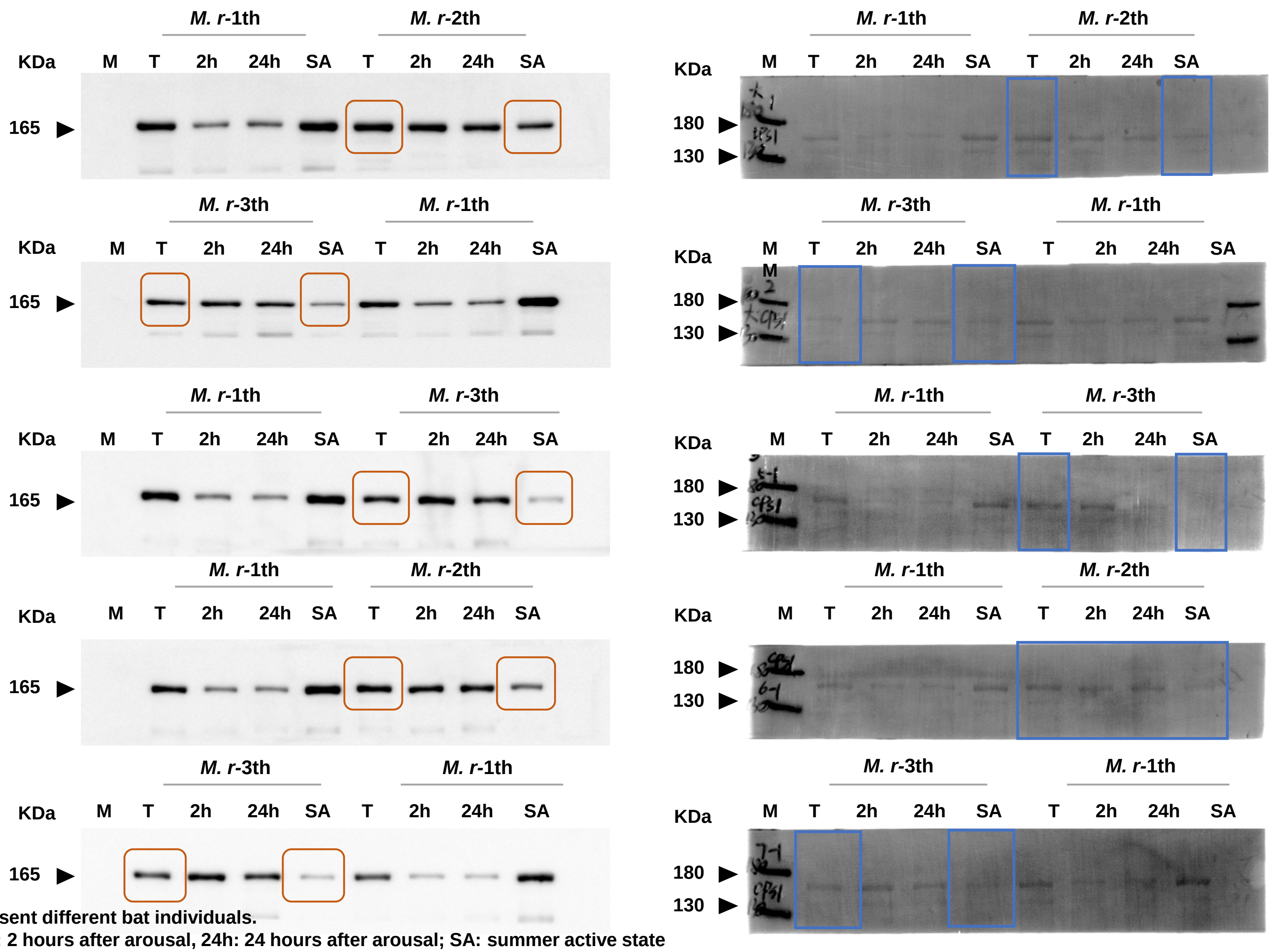

*Different numbers represent different bat individuals.
*M: marker, T: torpor, 2h: 2 hours after arousal, 24h: 24 hours after arousal; SA: summer active state

CPS1

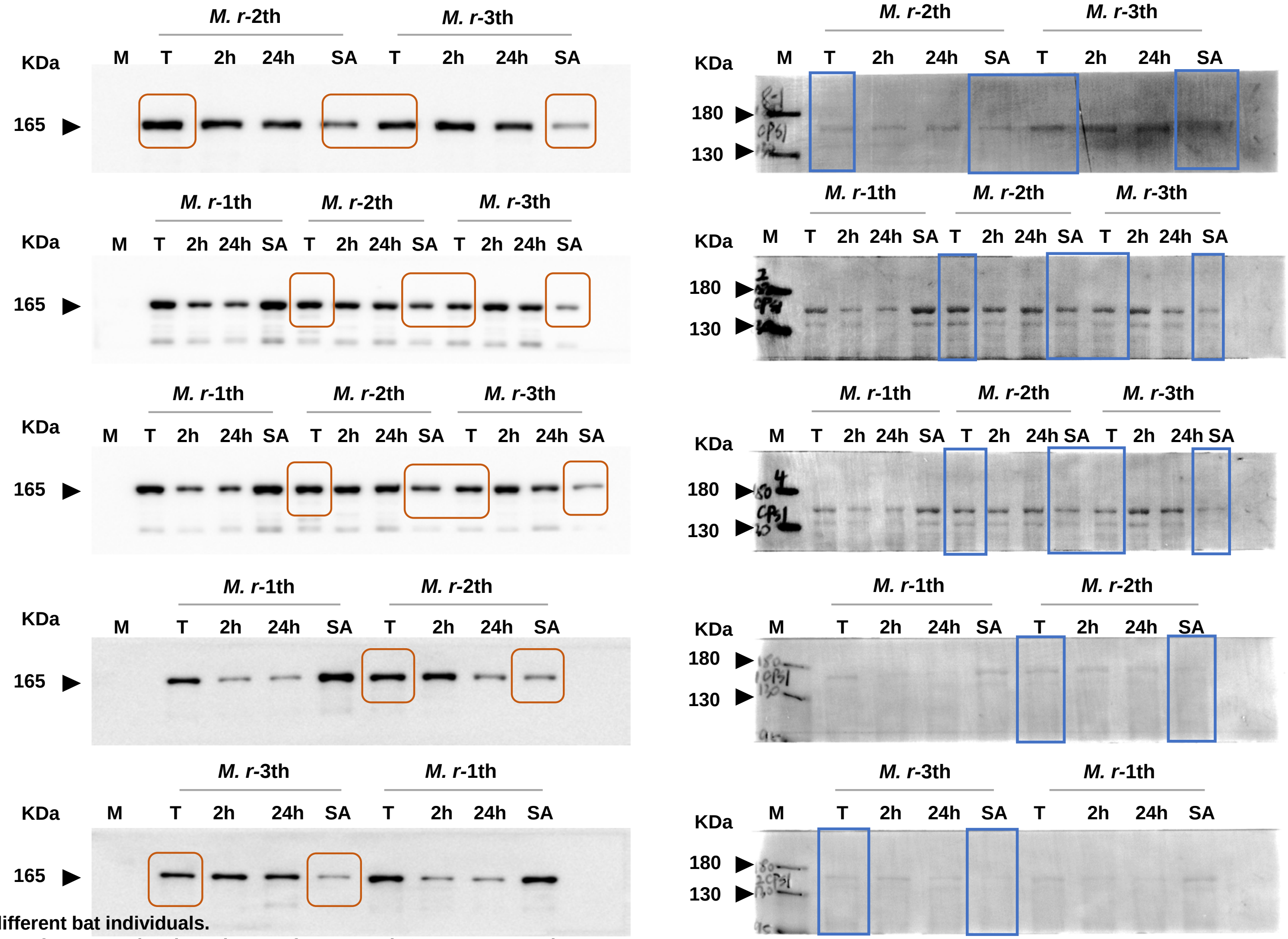

*Different numbers represent different bat individuals.
*M: marker, T: torpor, 2h: 2 hours after arousal, 24h: 24 hours after arousal; SA: summer active state

CPS1

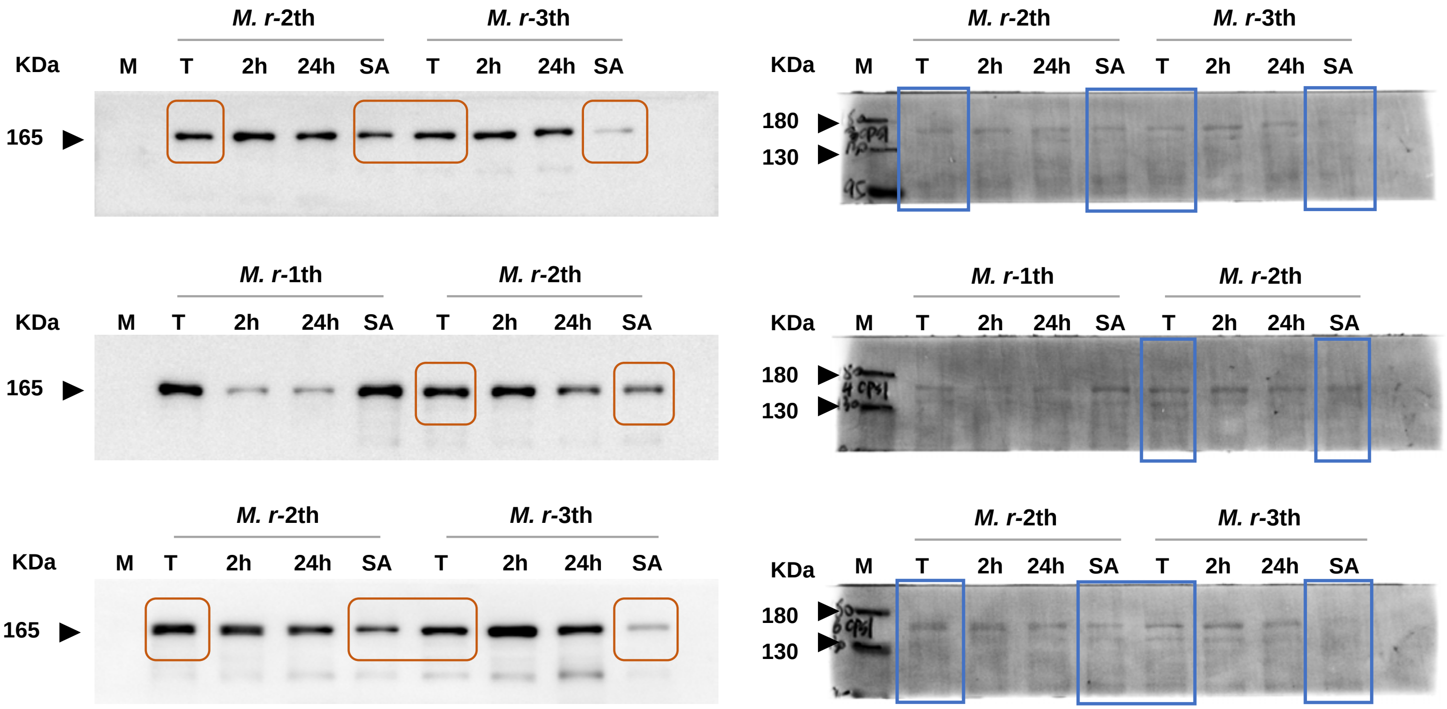

*Different numbers represent different bat individuals.
*M: marker, T: torpor, 2h: 2 hours after arousal, 24h: 24 hours after arousal; SA: summer active state

OTC

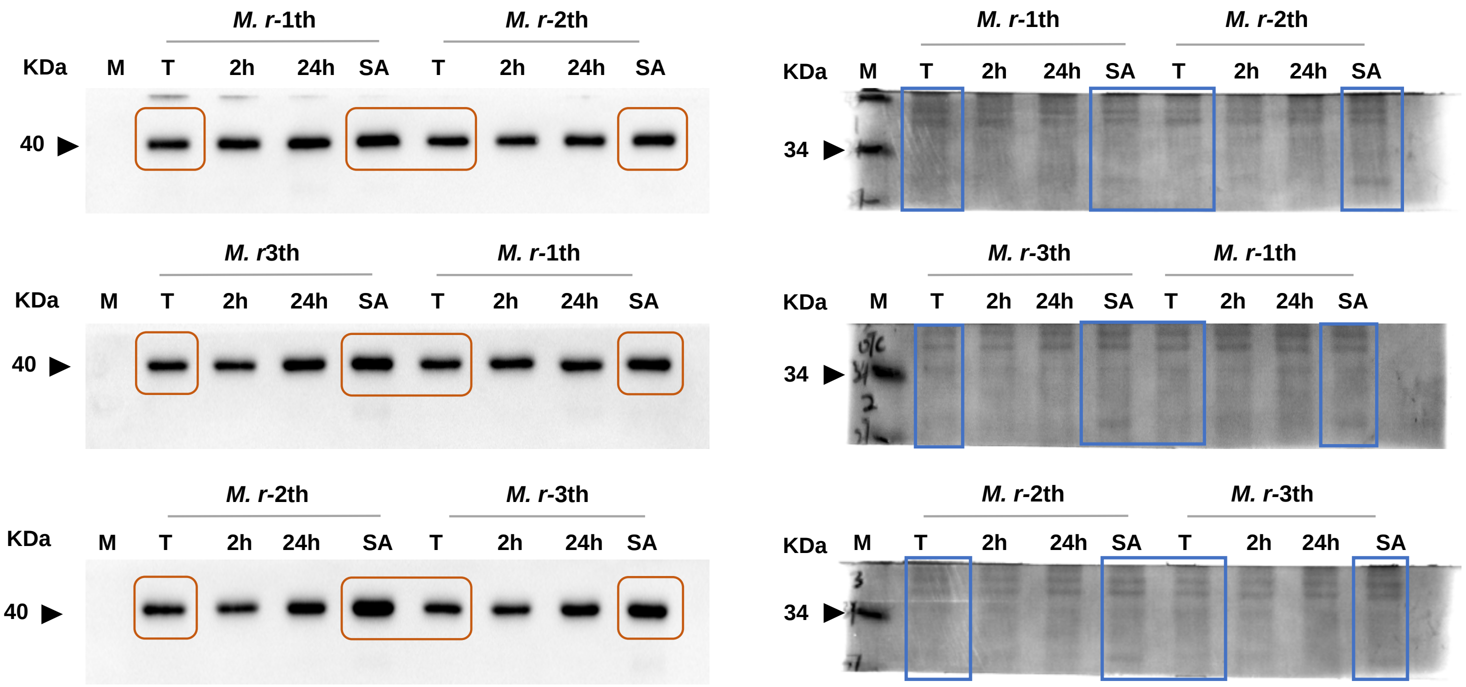

***Different numbers represent different bat individuals.**
***M: marker, T: torpor, 2h: 2 hours after arousal, 24h: 24 hours after arousal; SA: summer active state**

OTC

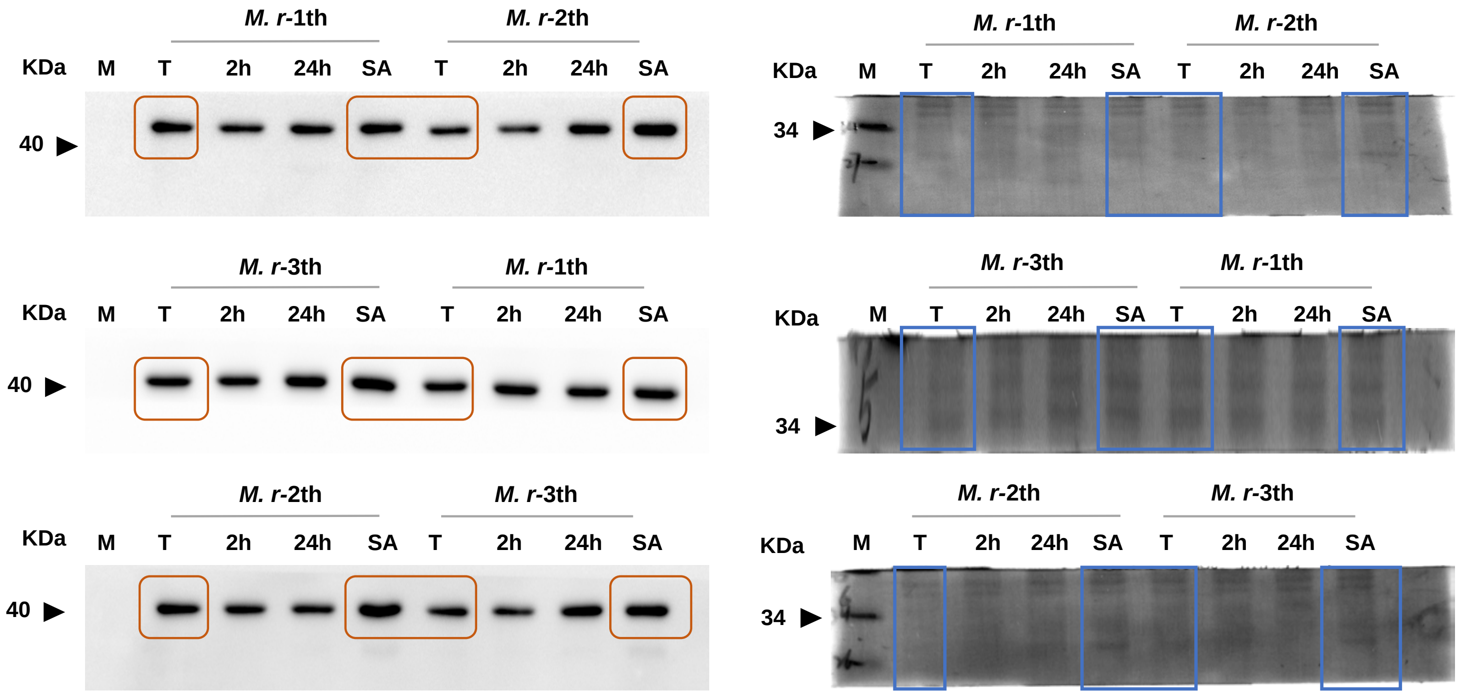

***Different numbers represent different bat individuals.**
***M: marker, T: torpor, 2h: 2 hours after arousal, 24h: 24 hours after arousal; SA: summer active state**

ASS1

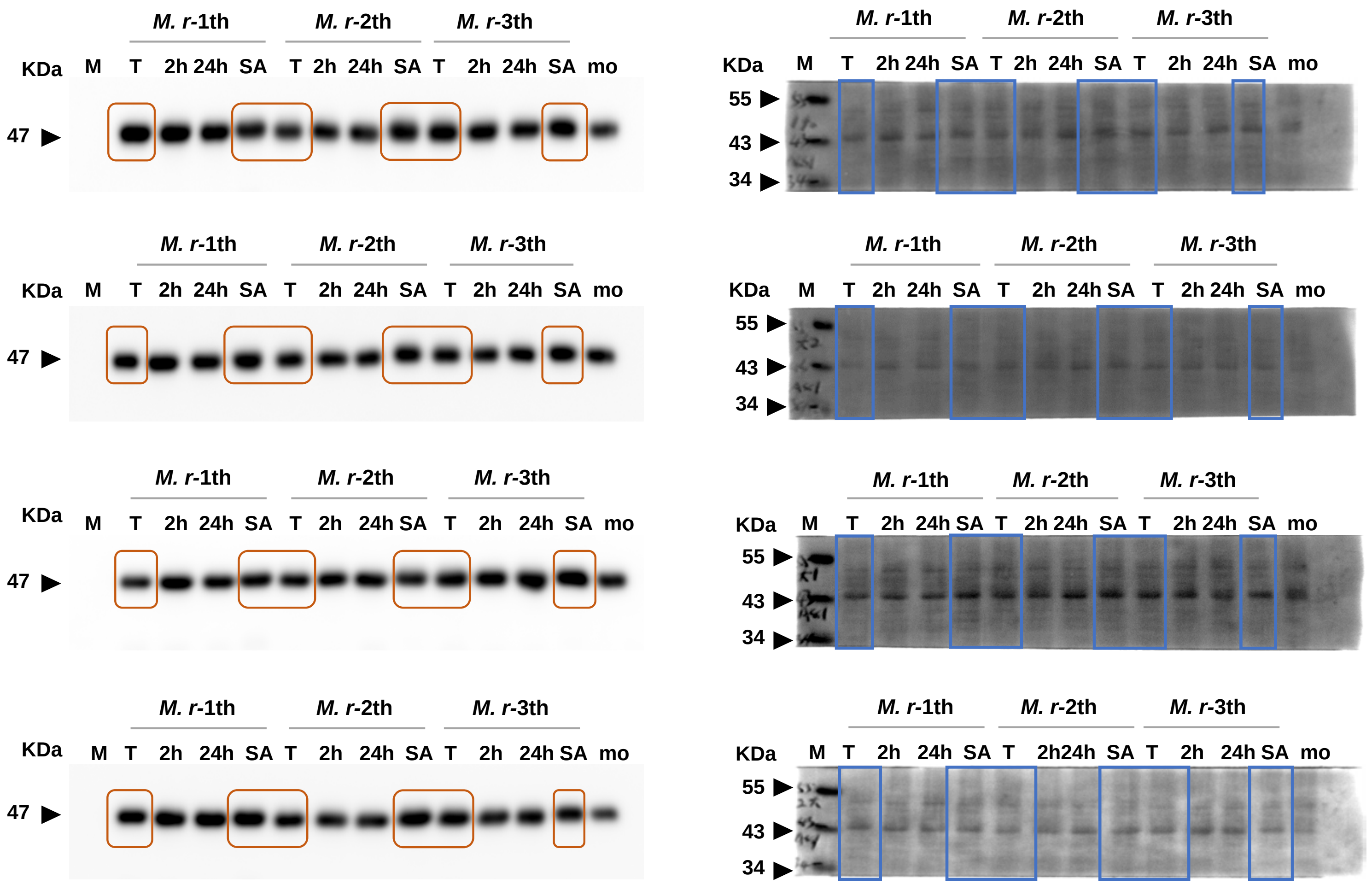

*Different numbers represent different bat individuals.
*M: marker, T: torpor, 2h: 2 hours after arousal, 24h: 24 hours after arousal; SA: summer active state
*mo: samples from mouse

ASS1

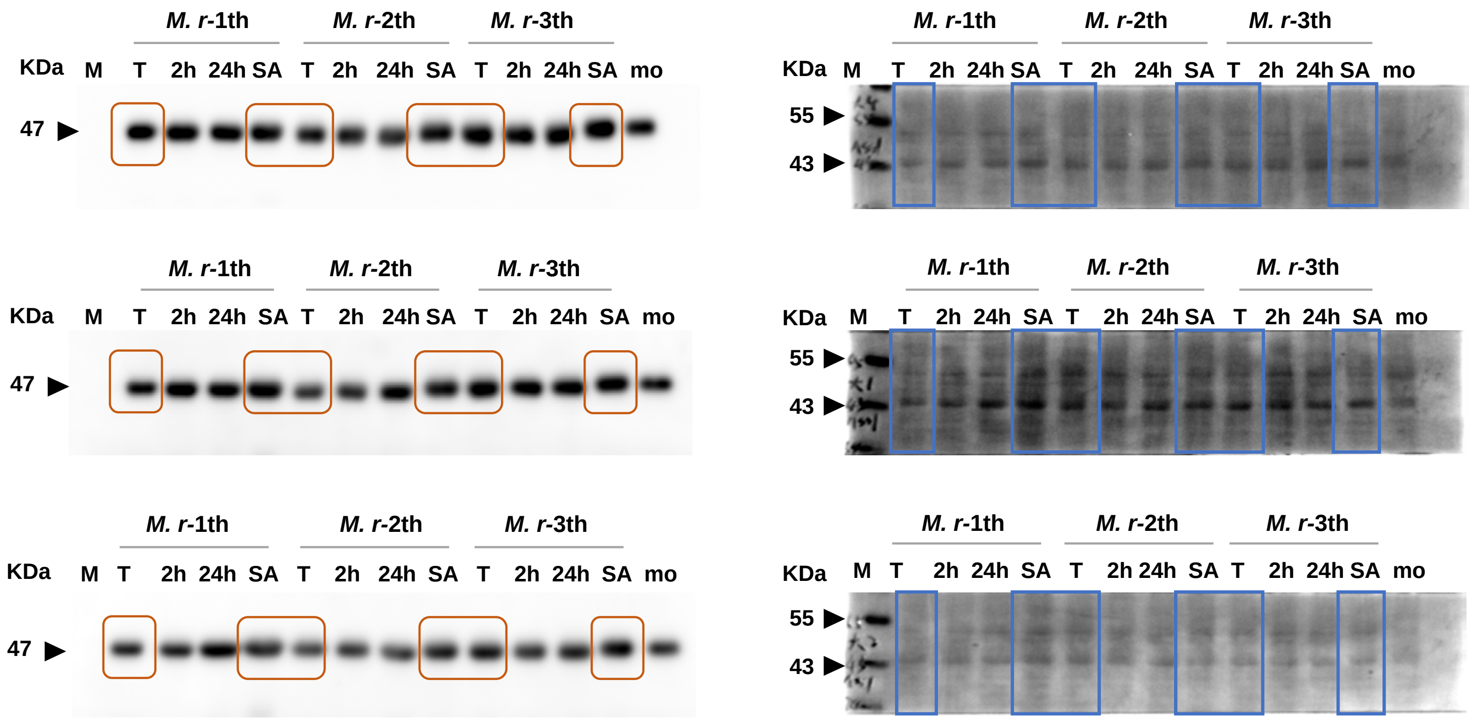

*Different numbers represent different bat individuals.
*M: marker, T: torpor, 2h: 2 hours after arousal, 24h: 24 hours after arousal; SA: summer active state
*mo: samples from mouse

ASL

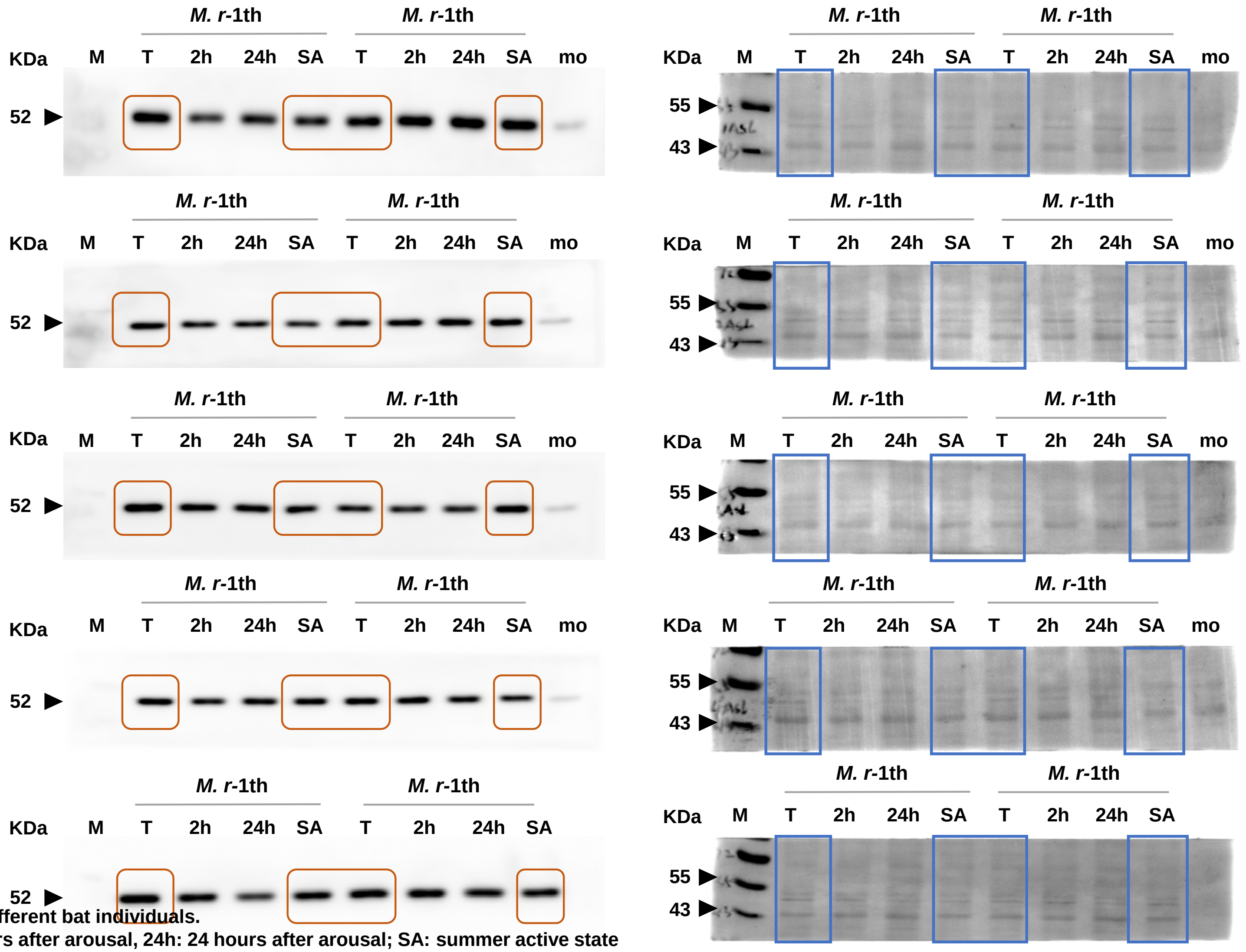

*Different numbers represent different bat individuals.
*M: marker, T: torpor, 2h: 2 hours after arousal, 24h: 24 hours after arousal; SA: summer active state
*mo: samples from mouse

ASL

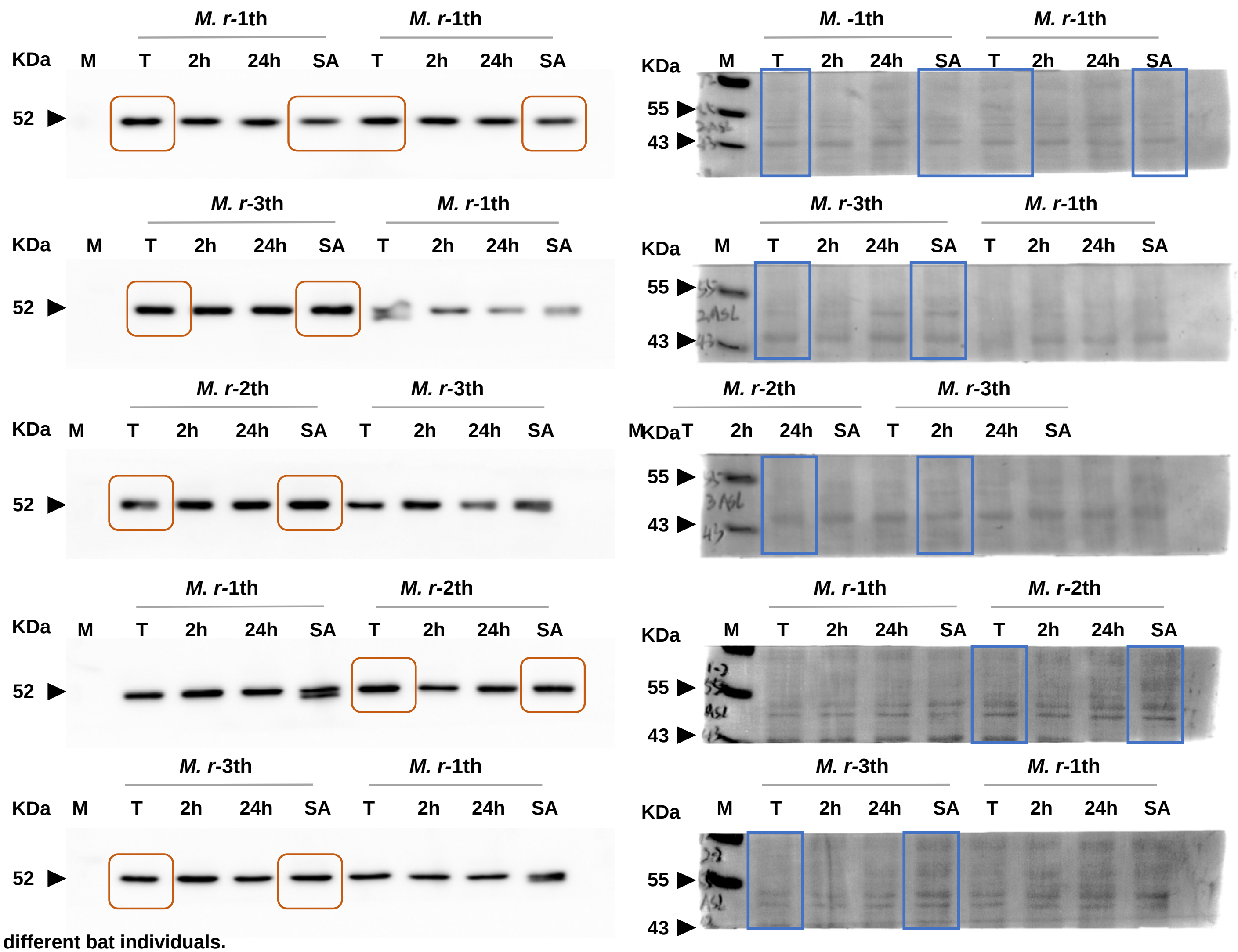

*Different numbers represent different bat individuals.
*M: marker, T: torpor, 2h: 2 hours after arousal, 24h: 24 hours after arousal; SA: summer active state

ASL

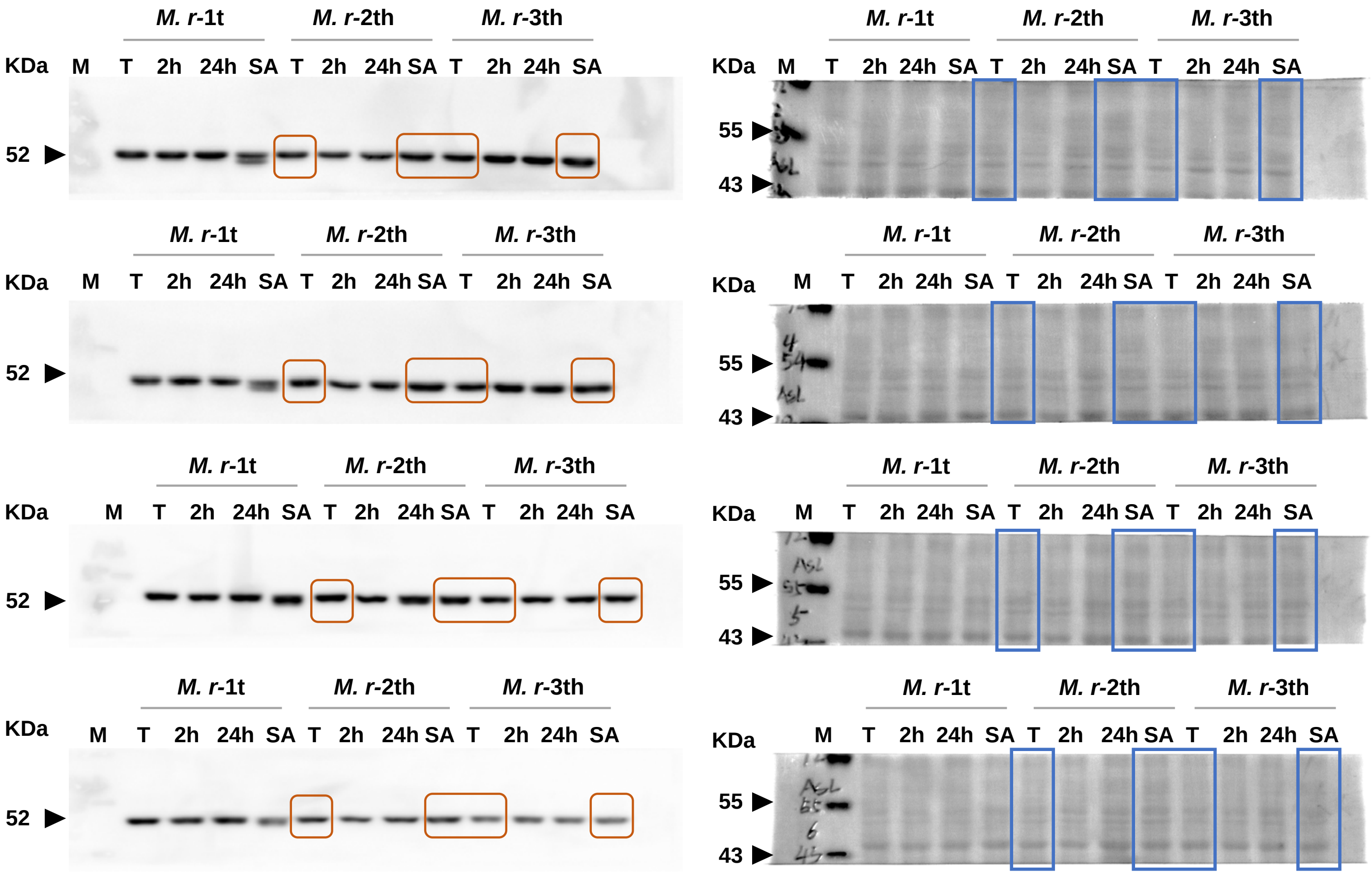

*Different numbers represent different bat individuals.
*M: marker, T: torpor, 2h: 2 hours after arousal, 24h: 24 hours after arousal; SA: summer active state

ARG

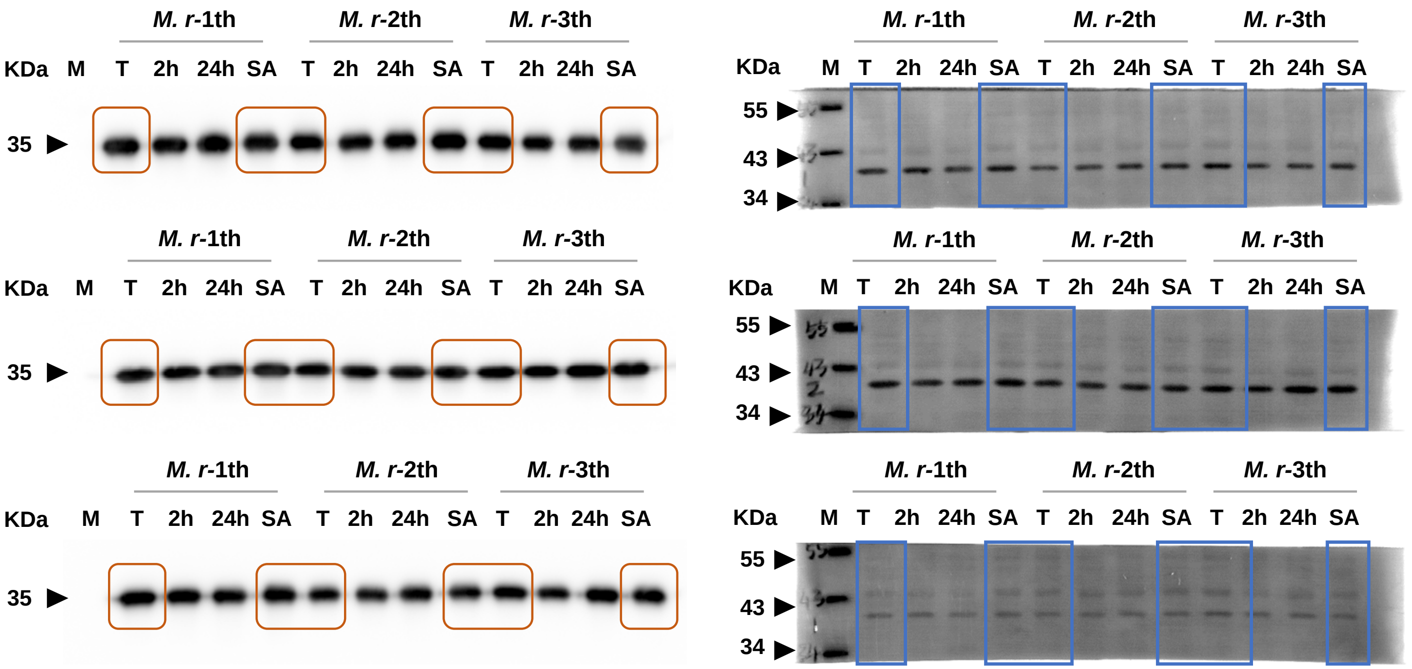

*Different numbers represent different bat individuals.
*M: marker, T: torpor, 2h: 2 hours after arousal, 24h: 24 hours after arousal; SA: summer active state

ARG

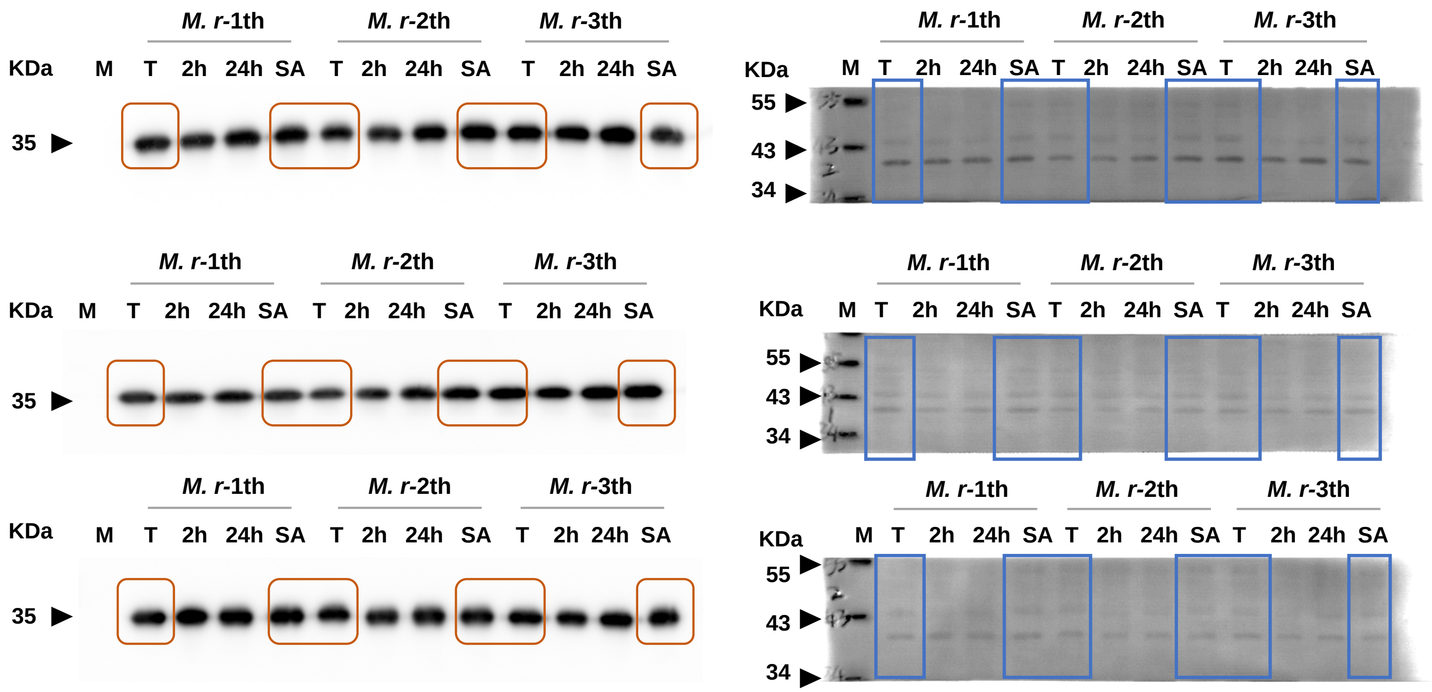

***Different numbers represent different bat individuals.**
***M: marker, T: torpor, 2h: 2 hours after arousal, 24h: 24 hours after arousal; SA: summer active state**

ODC

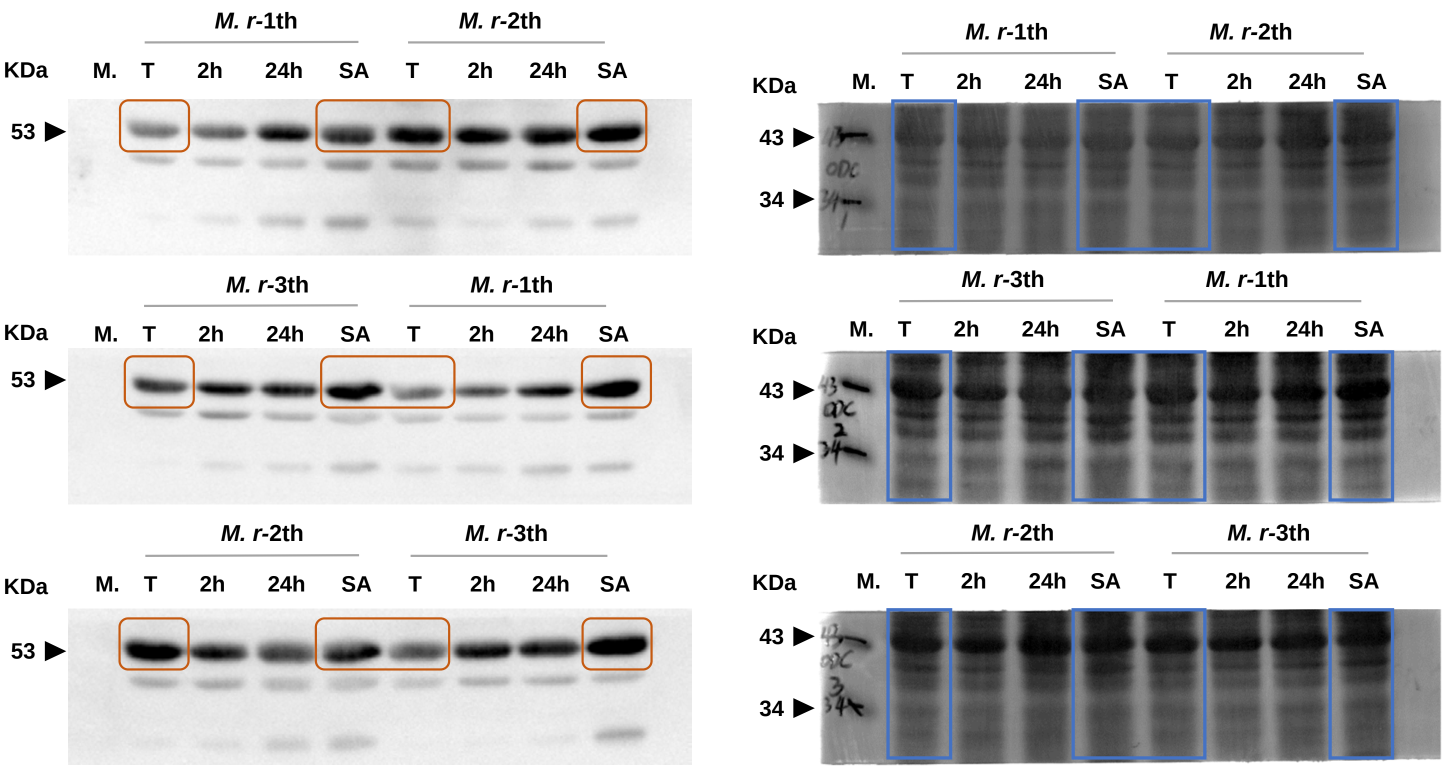

*Different numbers represent different bat individuals.
*M: marker, T: torpor, 2h: 2 hours after arousal, 24h: 24 hours after arousal; SA: summer active state

ODC

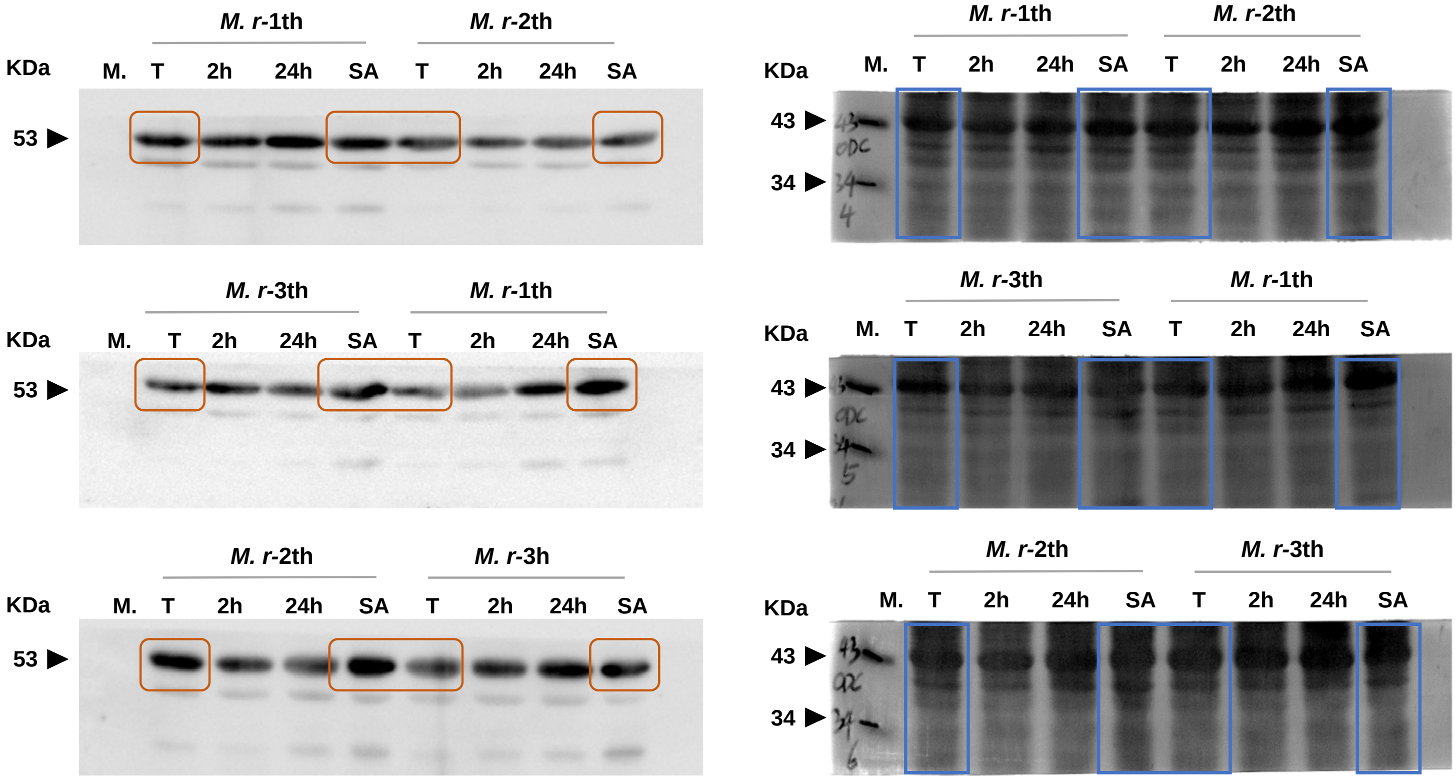

***Different numbers represent different bat individuals.**
***M: marker, T: torpor, 2h: 2 hours after arousal, 24h: 24 hours after arousal; SA: summer active state**

AGMAT

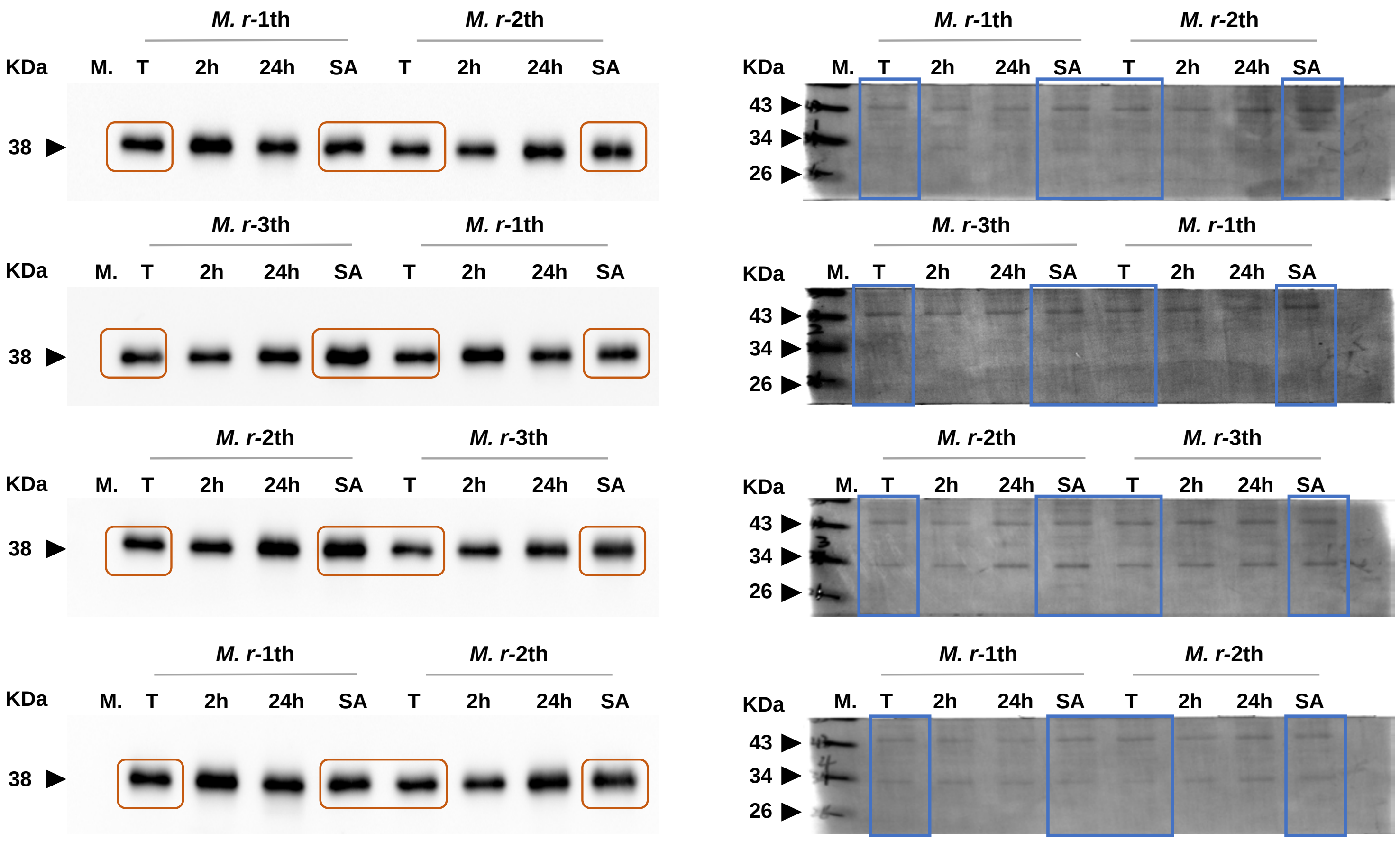

*Different numbers represent different bat individuals.
*M: marker, T: torpor, 2h: 2 hours after arousal, 24h: 24 hours after arousal; SA: summer active state

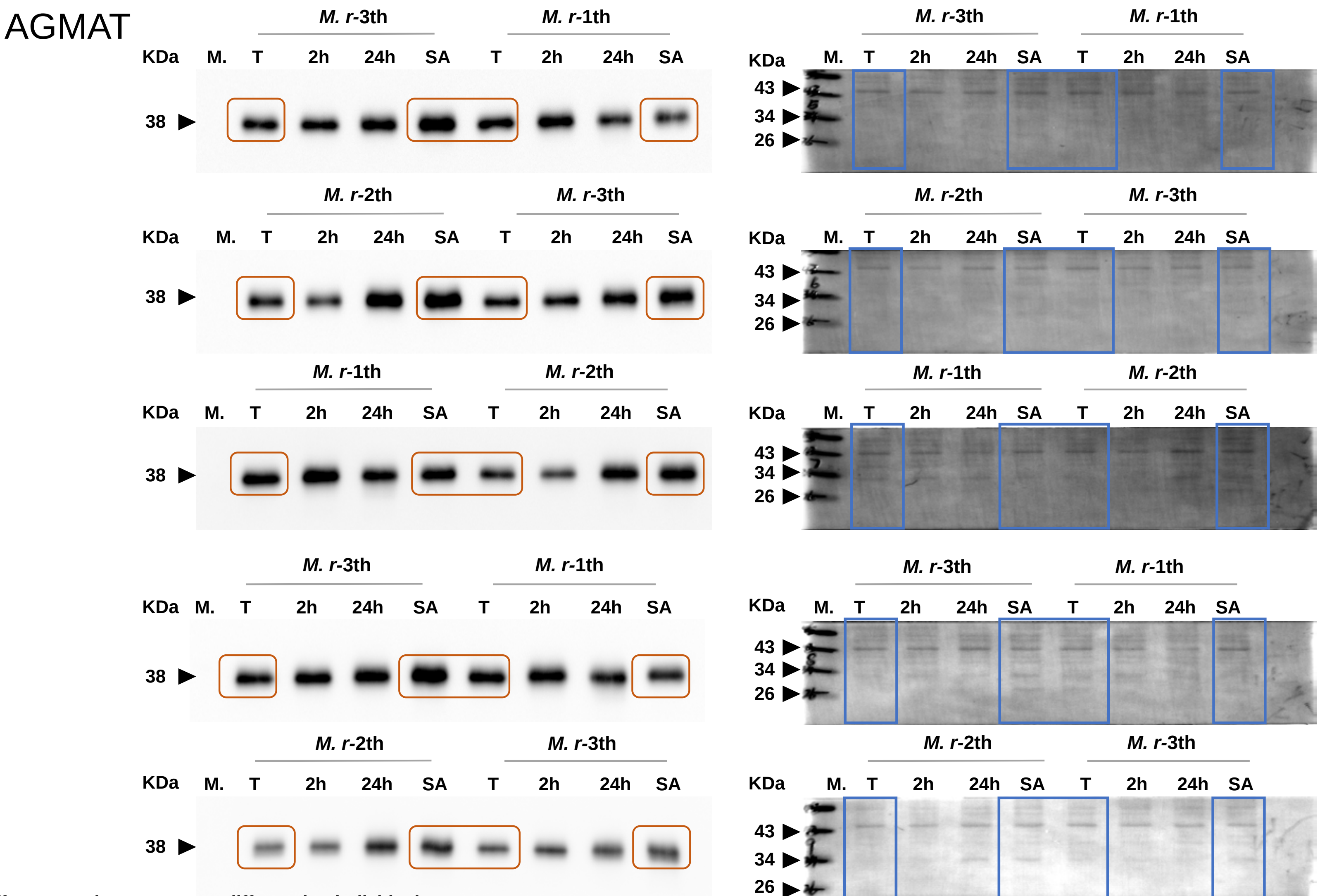

*Different numbers represent different bat individuals.
*M: marker, T: torpor, 2h: 2 hours after arousal, 24h: 24 hours after arousal; SA: summer active state

NAGS

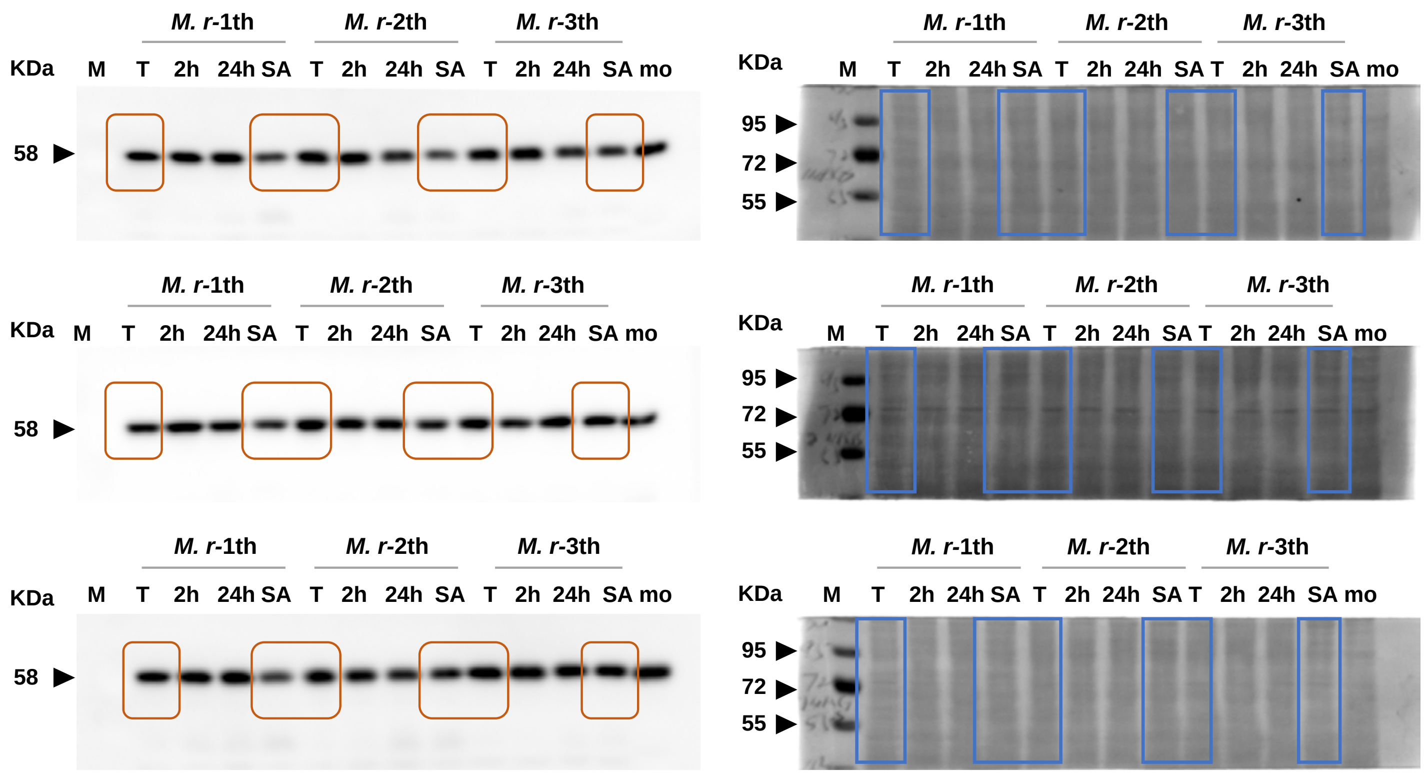

*Different numbers represent different bat individuals.
*M: marker, T: torpor, 2h: 2 hours after arousal, 24h: 24 hours after arousal; SA: summer active state
*mo: samples from mouse

NAGS

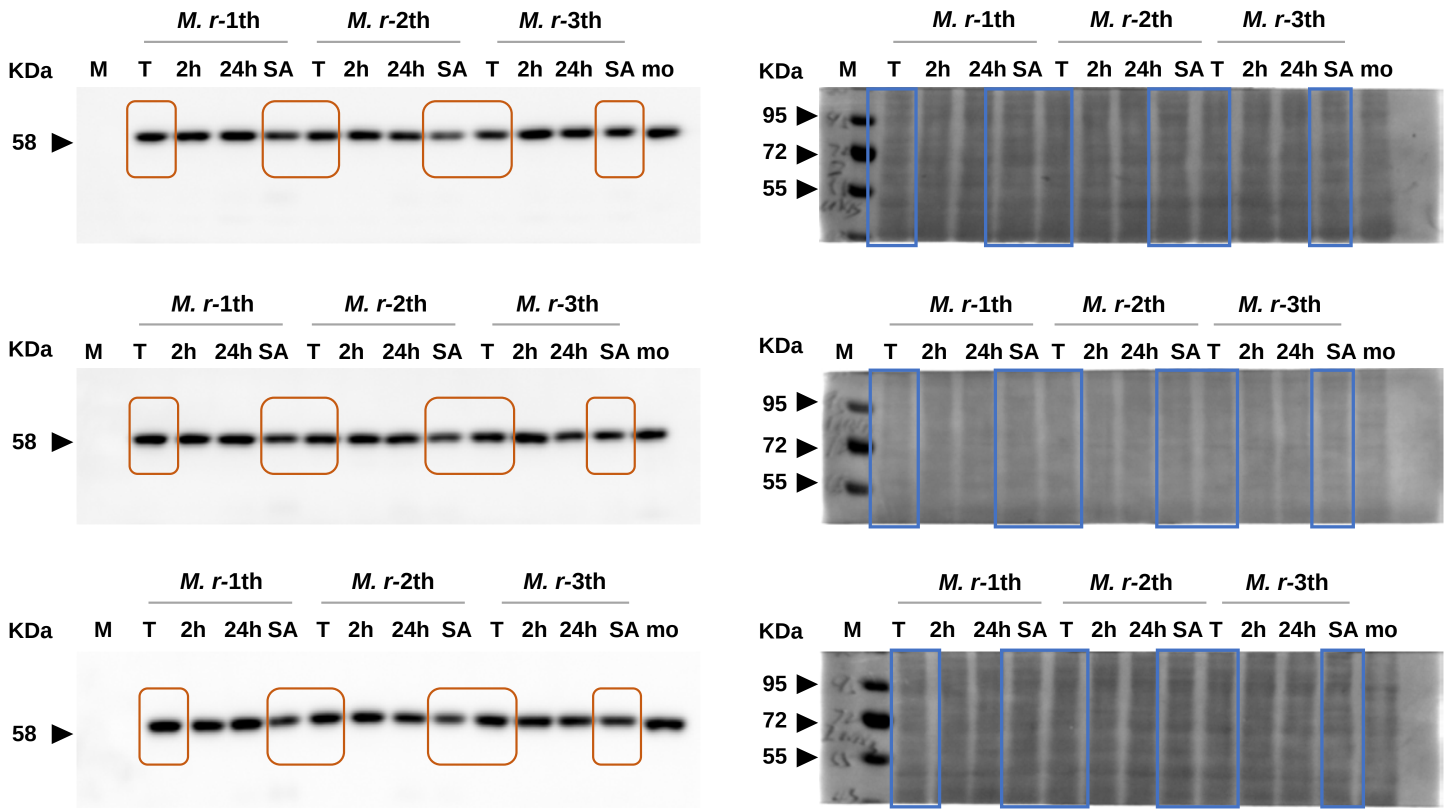

*Different numbers represent different bat individuals.
*M: marker, T: torpor, 2h: 2 hours after arousal, 24h: 24 hours after arousal; SA: summer active state
*mo: samples from mouse

SIRT5

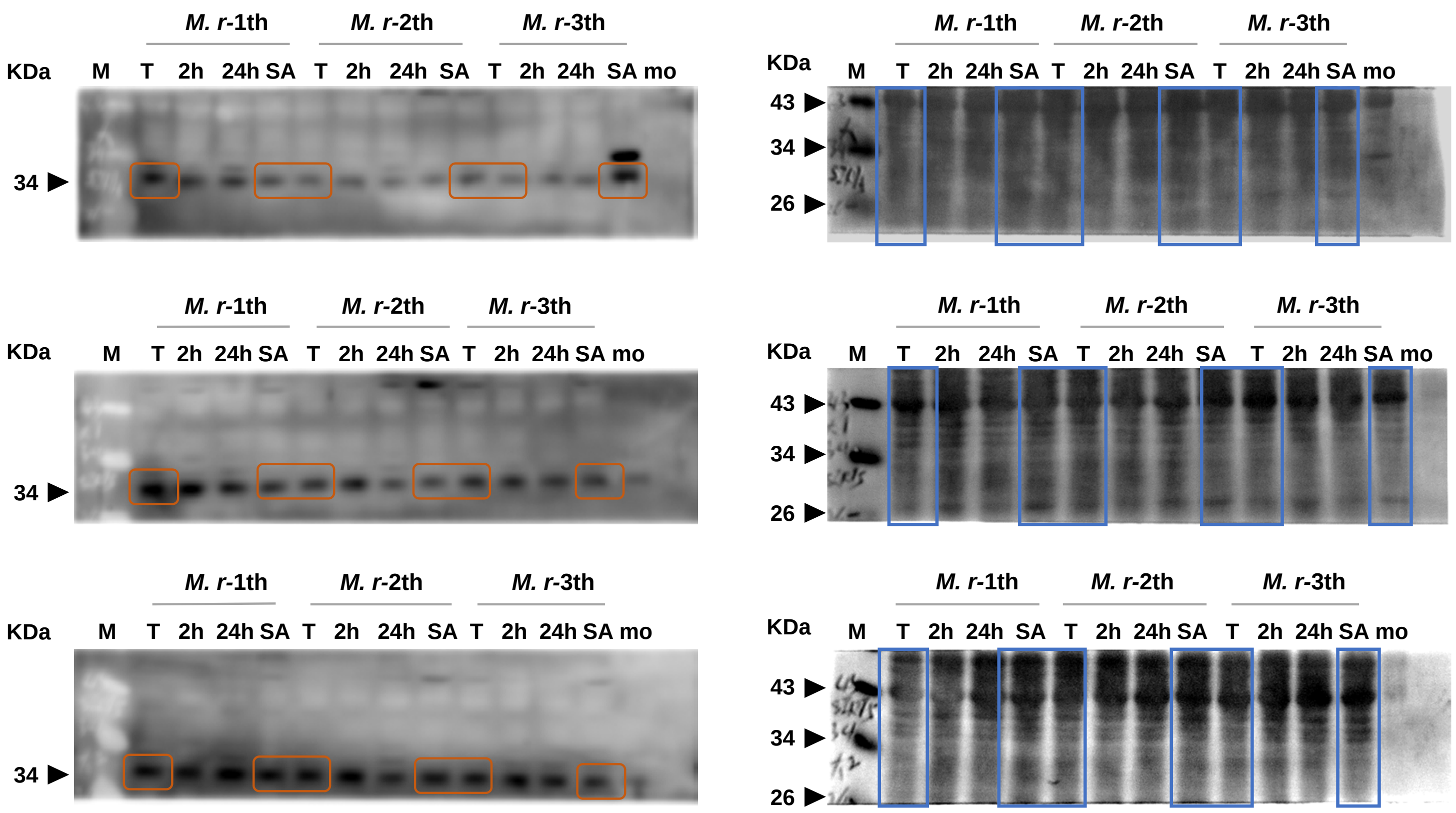

*Different numbers represent different bat individuals.
*M: marker, T: torpor, 2h: 2 hours after arousal, 24h: 24 hours after arousal; SA: summer active state
*mo: samples from mouse

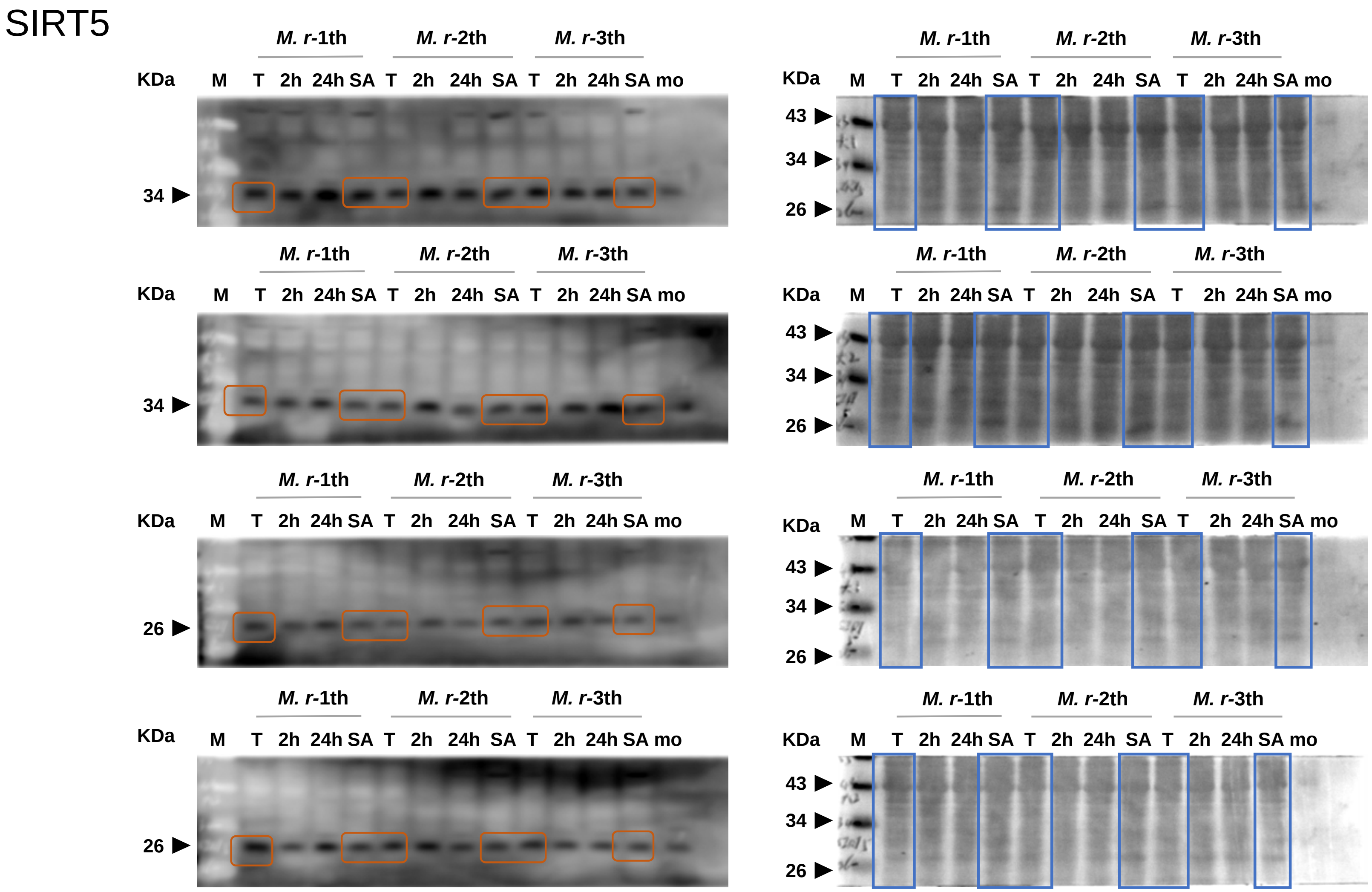

*Different numbers represent different bat individuals.
*M: marker, T: torpor, 2h: 2 hours after arousal, 24h: 24 hours after arousal; SA: summer active state
*mo: samples from mouse

Rhinolophus ferrumequinum

All WB images

CPS1

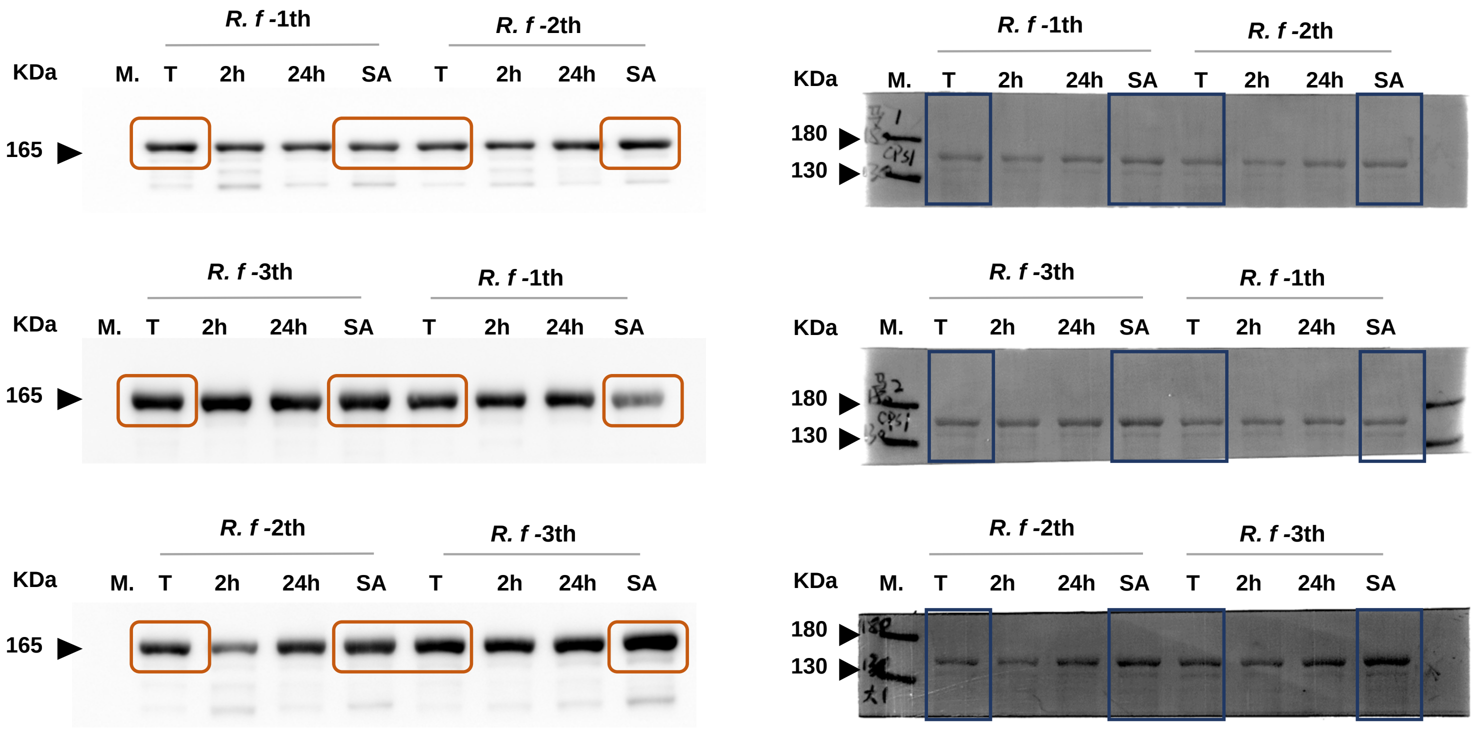

*Different numbers represent different bat individuals.
*M: marker, T: torpor, 2h: 2 hours after arousal, 24h: 24 hours after arousal; SA: summer active state

CPS1

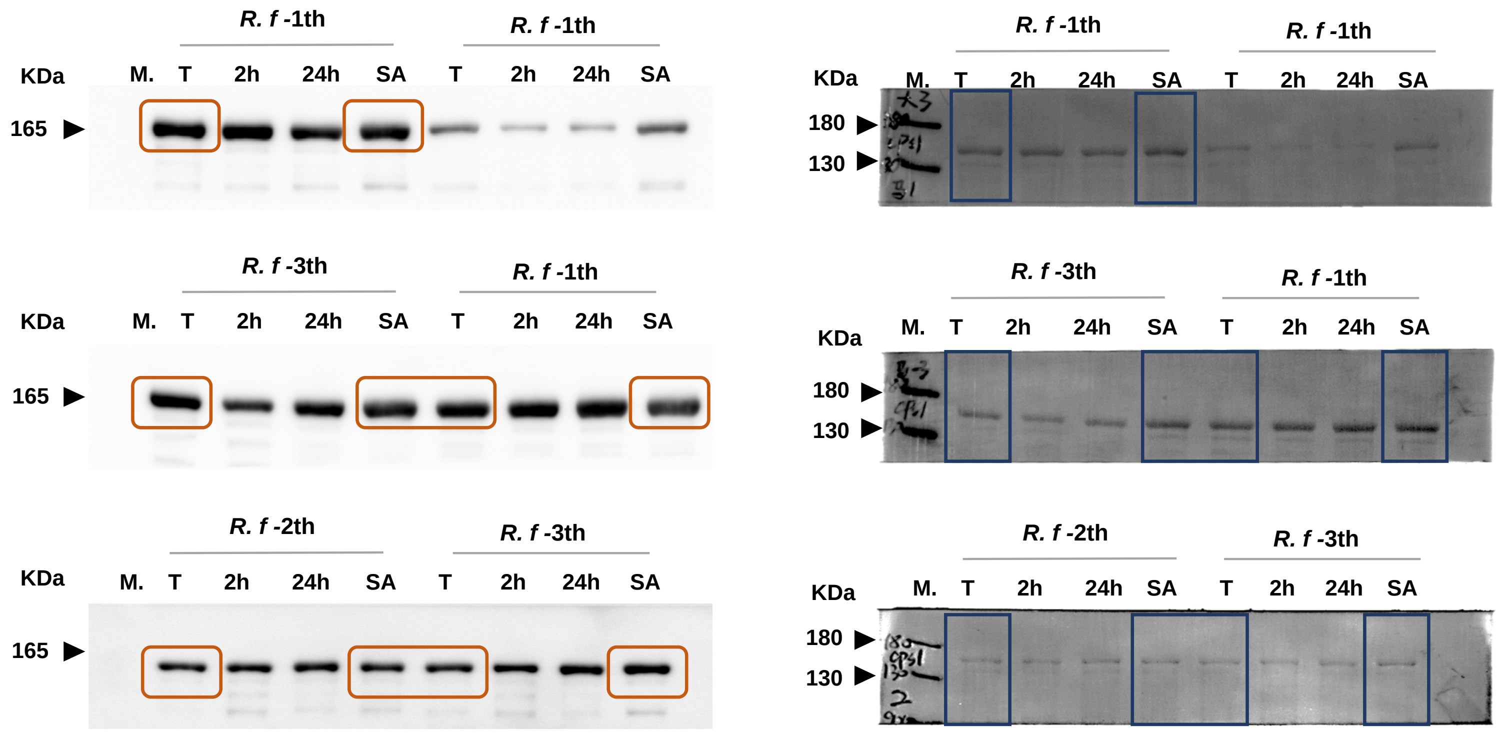

*Different numbers represent different bat individuals.

*M: marker, T: torpor, 2h: 2 hours after arousal, 24h: 24 hours after arousal; SA: summer active state

OTC

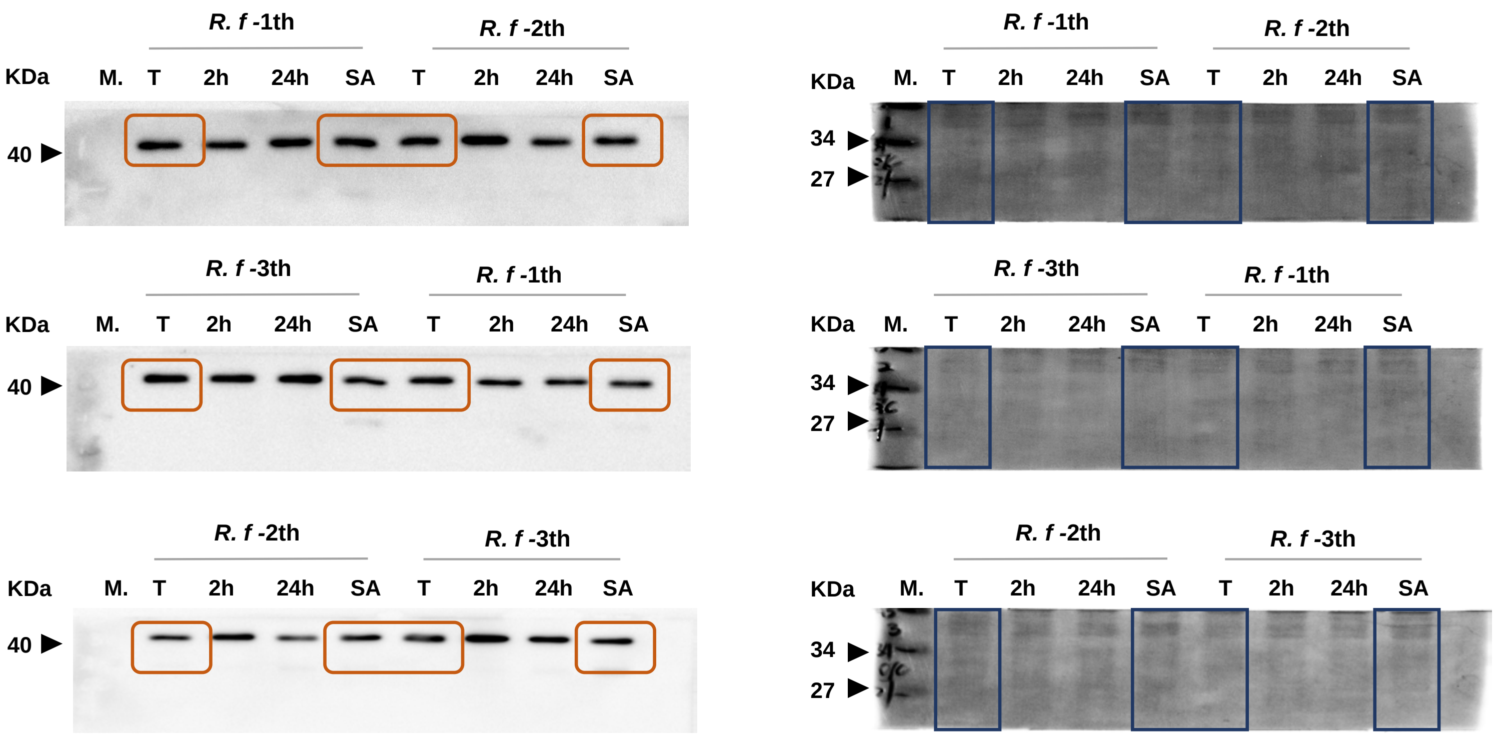

*Different numbers represent different bat individuals.
*M: marker, T: torpor, 2h: 2 hours after arousal, 24h: 24 hours after arousal; SA: summer active state

OTC

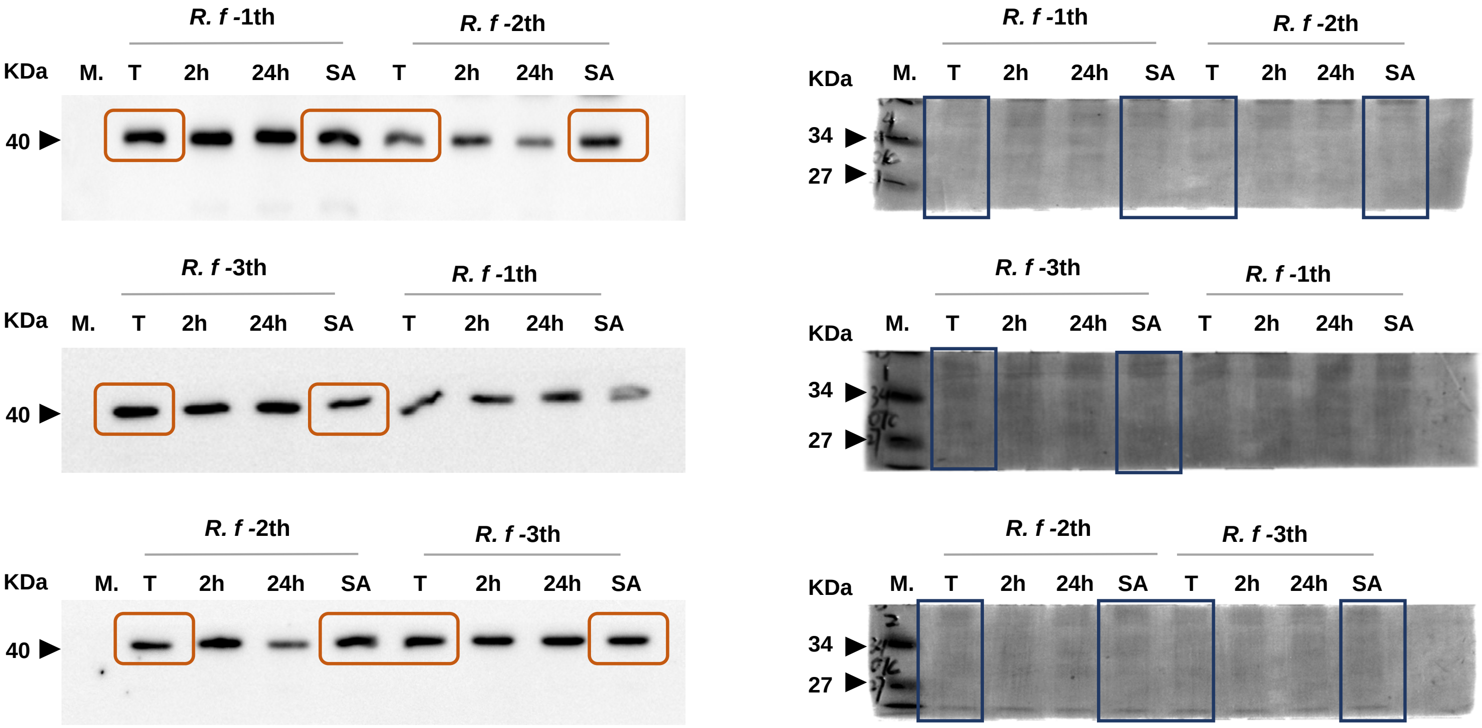

*Different numbers represent different bat individuals.
*M: marker, T: torpor, 2h: 2 hours after arousal, 24h: 24 hours after arousal; SA: summer active state

ASS1

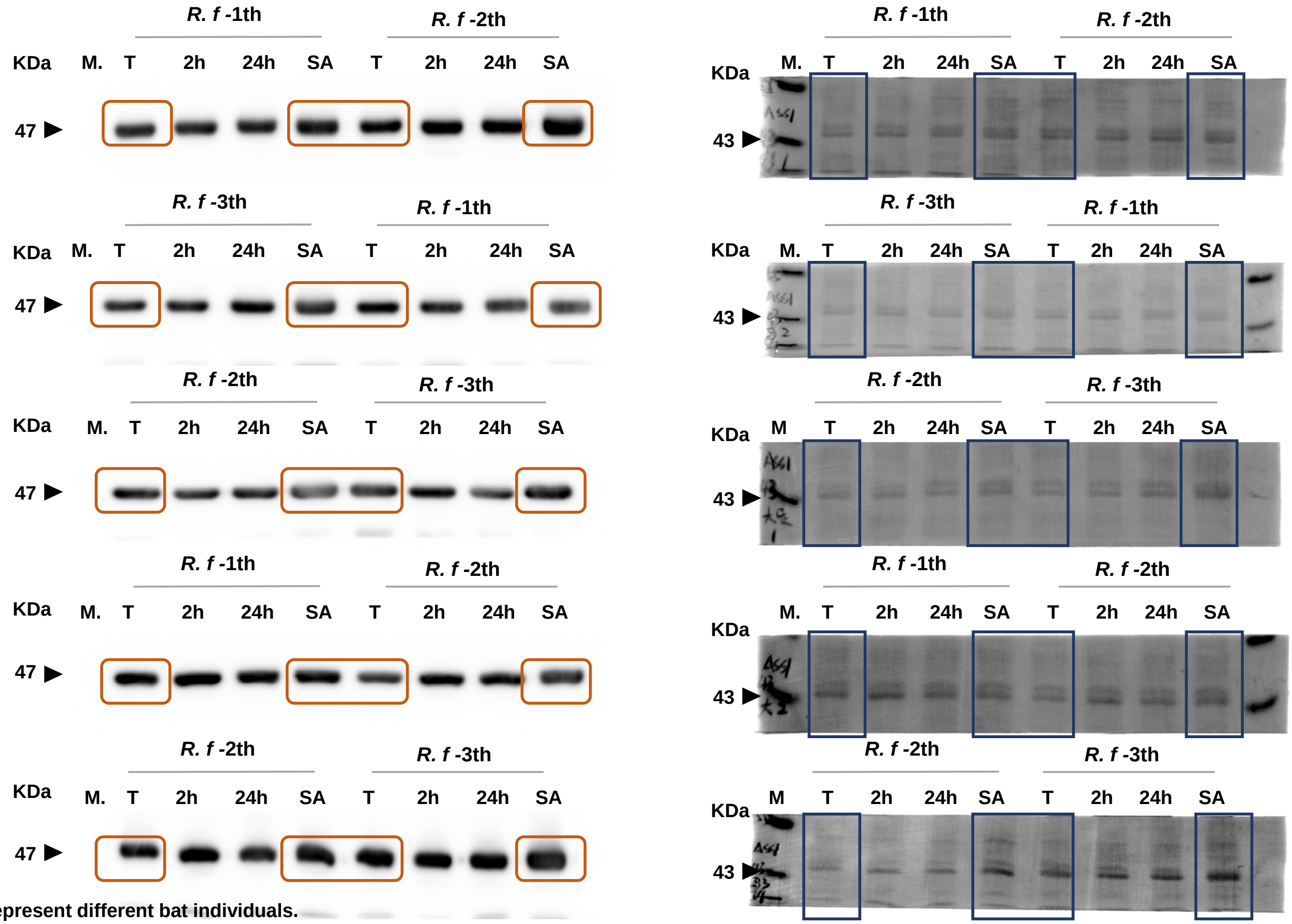

*Different numbers represent different bat individuals.
*M: marker, T: torpor, 2h: 2 hours after arousal, 24h: 24 hours after arousal; SA: summer active state

ASL

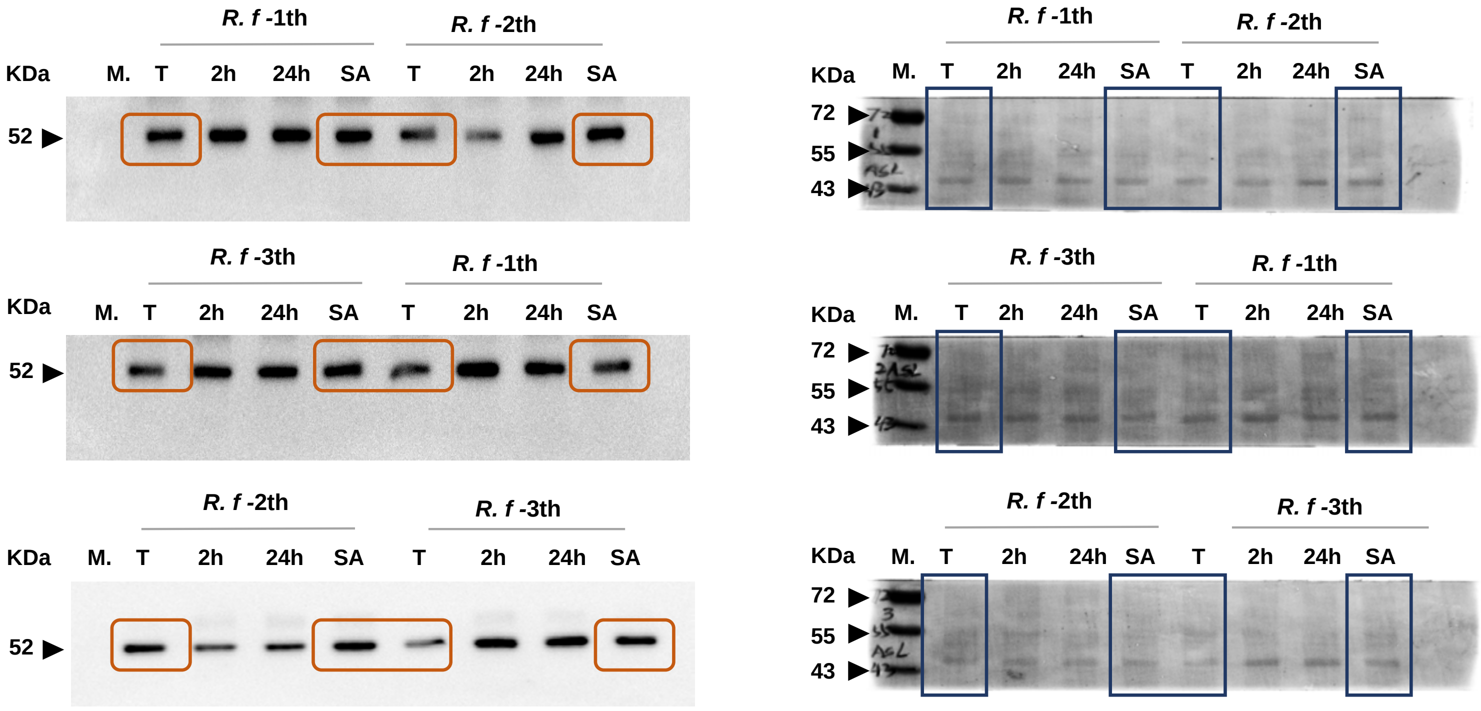

*Different numbers represent different bat individuals.
*M: marker, T: torpor, 2h: 2 hours after arousal, 24h: 24 hours after arousal; SA: summer active state

ASL

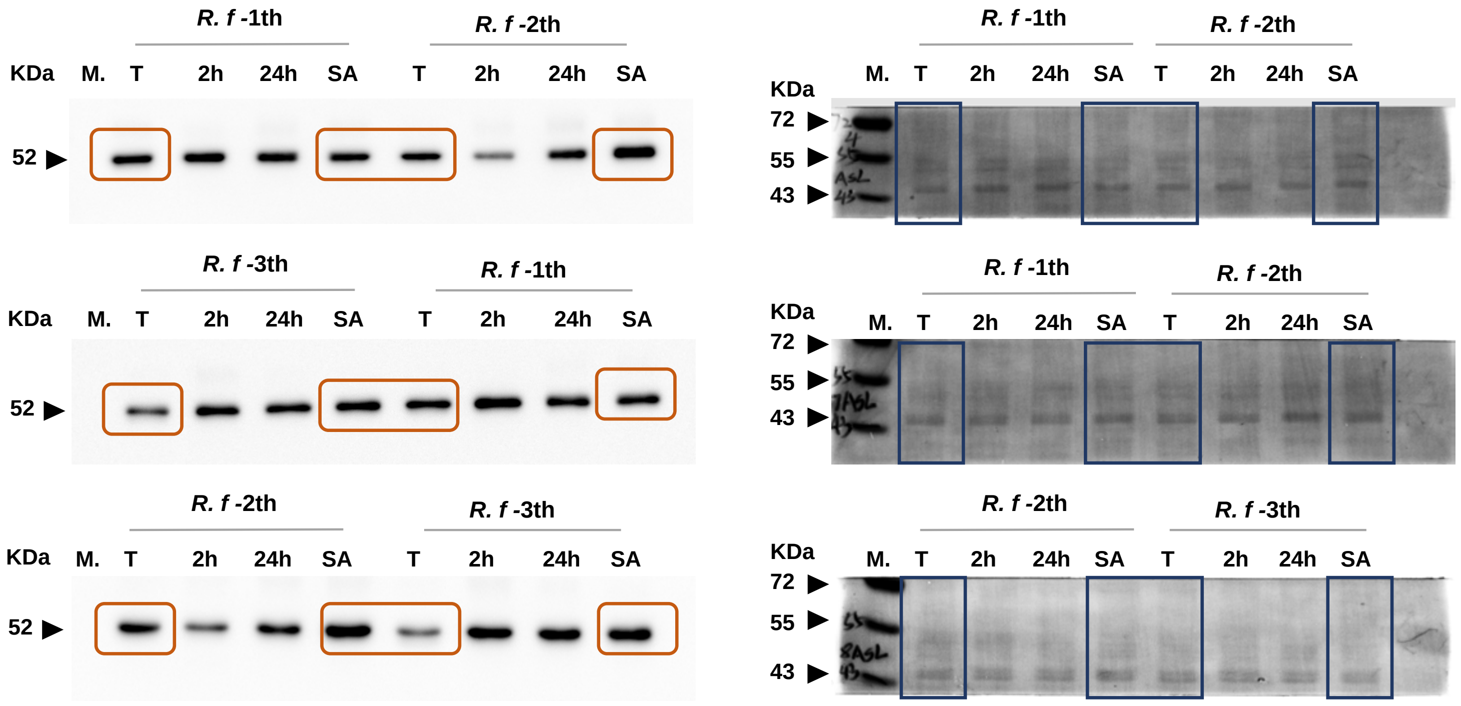

*Different numbers represent different bat individuals.
*M: marker, T: torpor, 2h: 2 hours after arousal, 24h: 24 hours after arousal; SA: summer active state

ARG

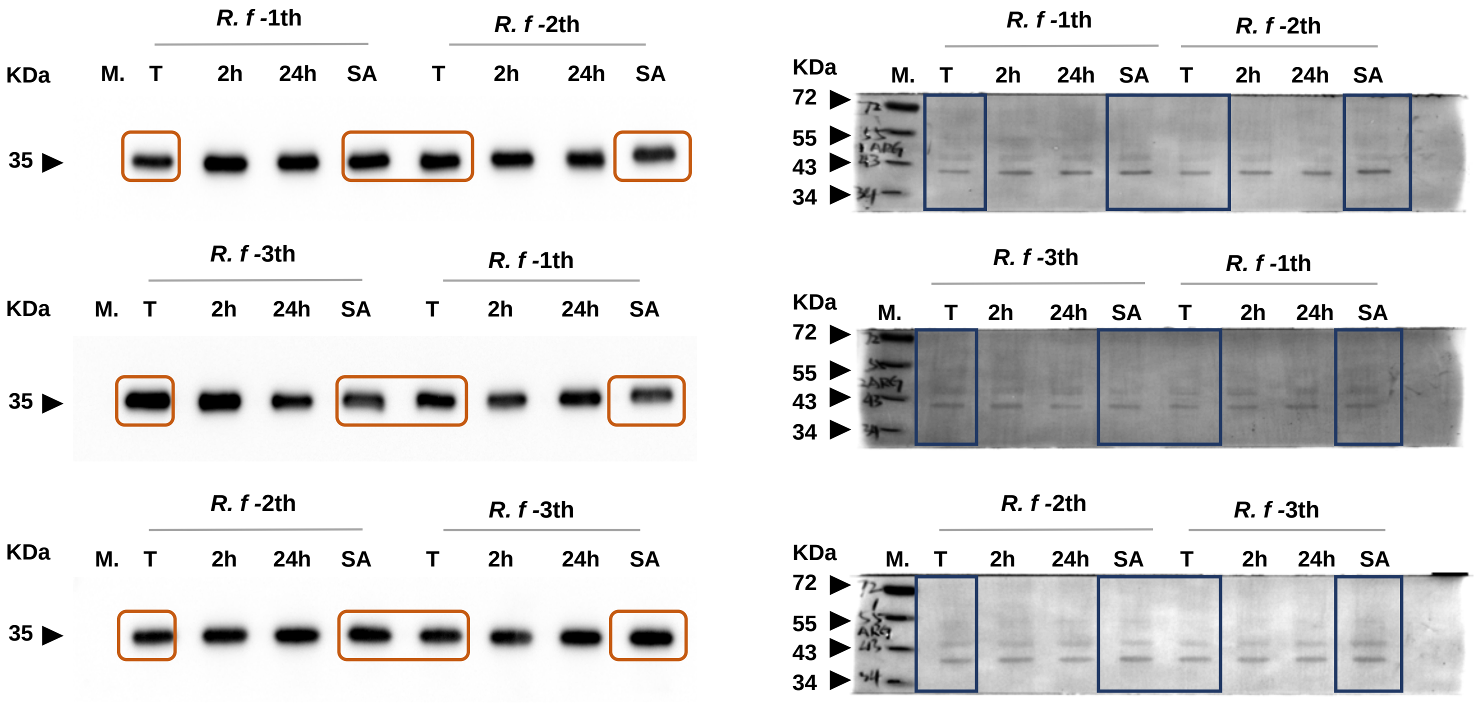

*Different numbers represent different bat individuals.
*M: marker, T: torpor, 2h: 2 hours after arousal, 24h: 24 hours after arousal; SA: summer active state

ARG

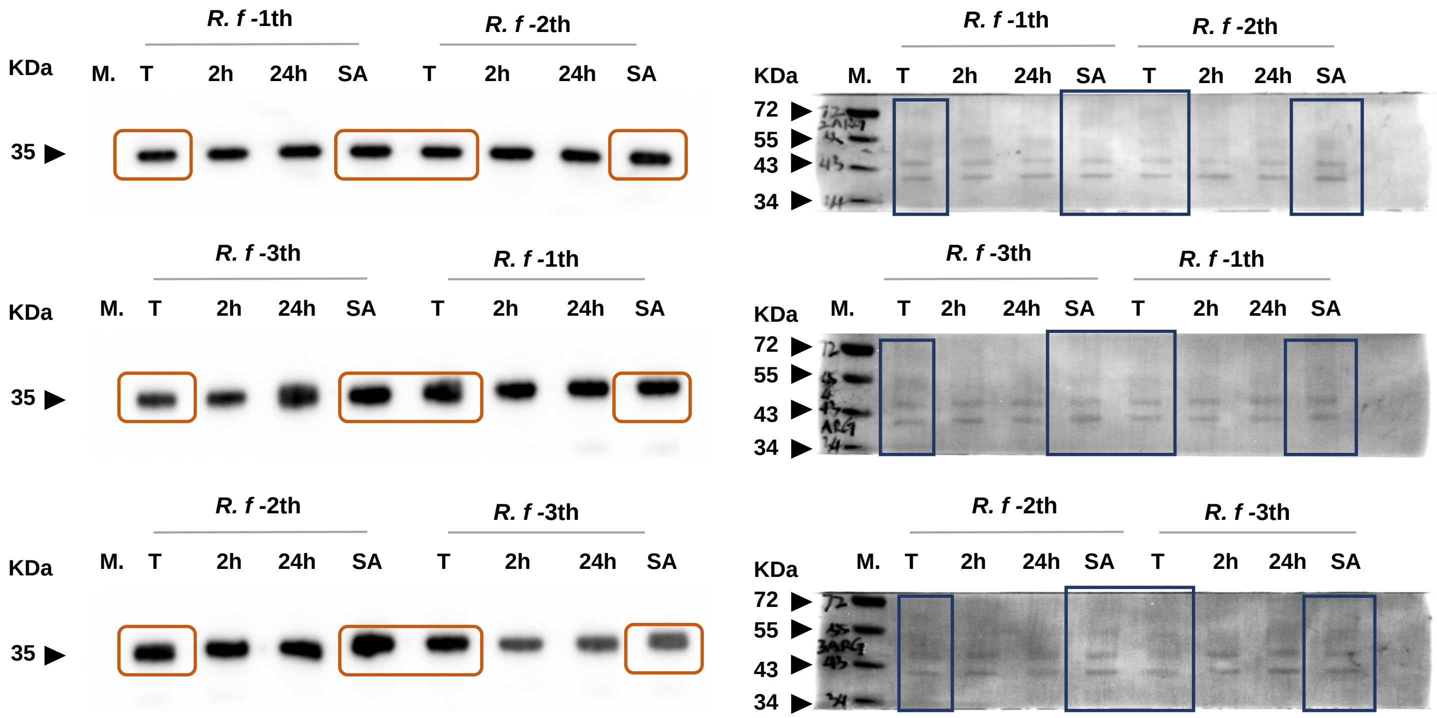

*Different numbers represent different bat individuals.
*M: marker, T: torpor, 2h: 2 hours after arousal, 24h: 24 hours after arousal; SA: summer active state

ODC

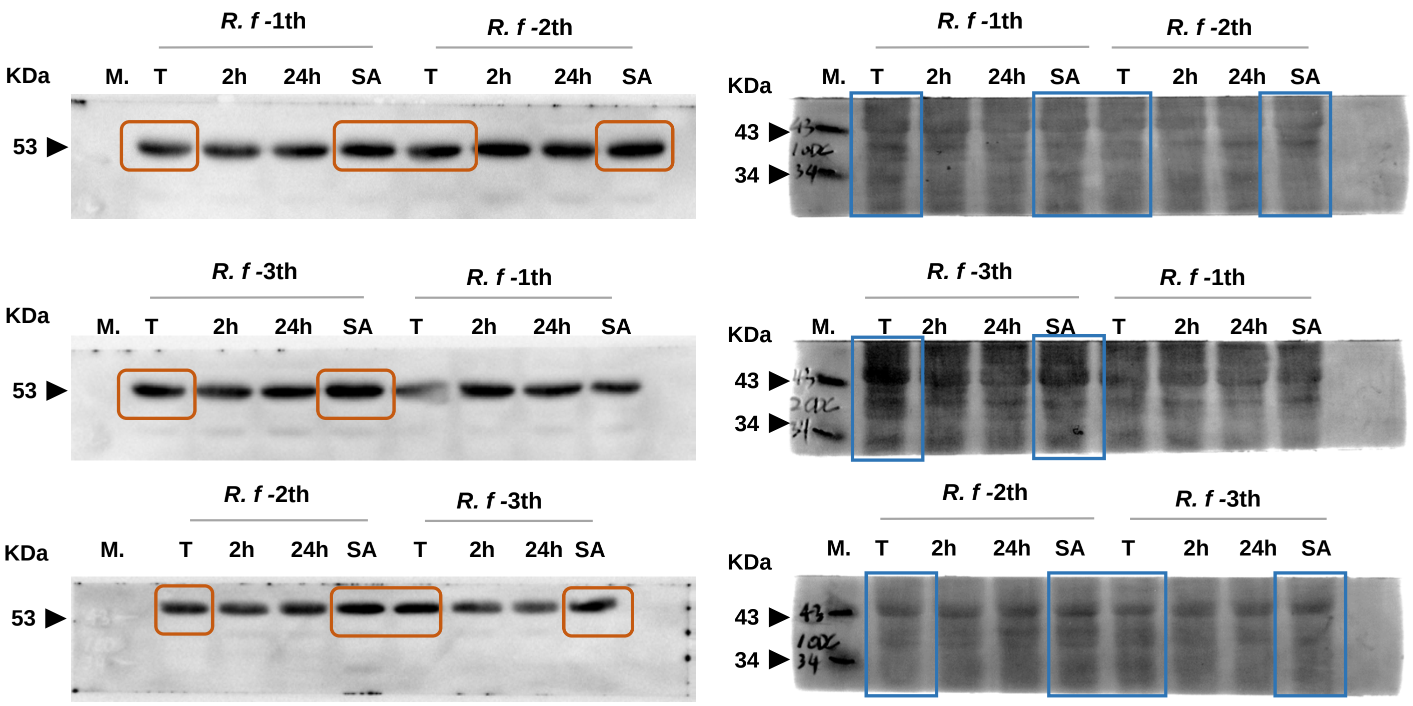

*Different numbers represent different bat individuals.
*M: marker, T: torpor, 2h: 2 hours after arousal, 24h: 24 hours after arousal; SA: summer active state

ODC

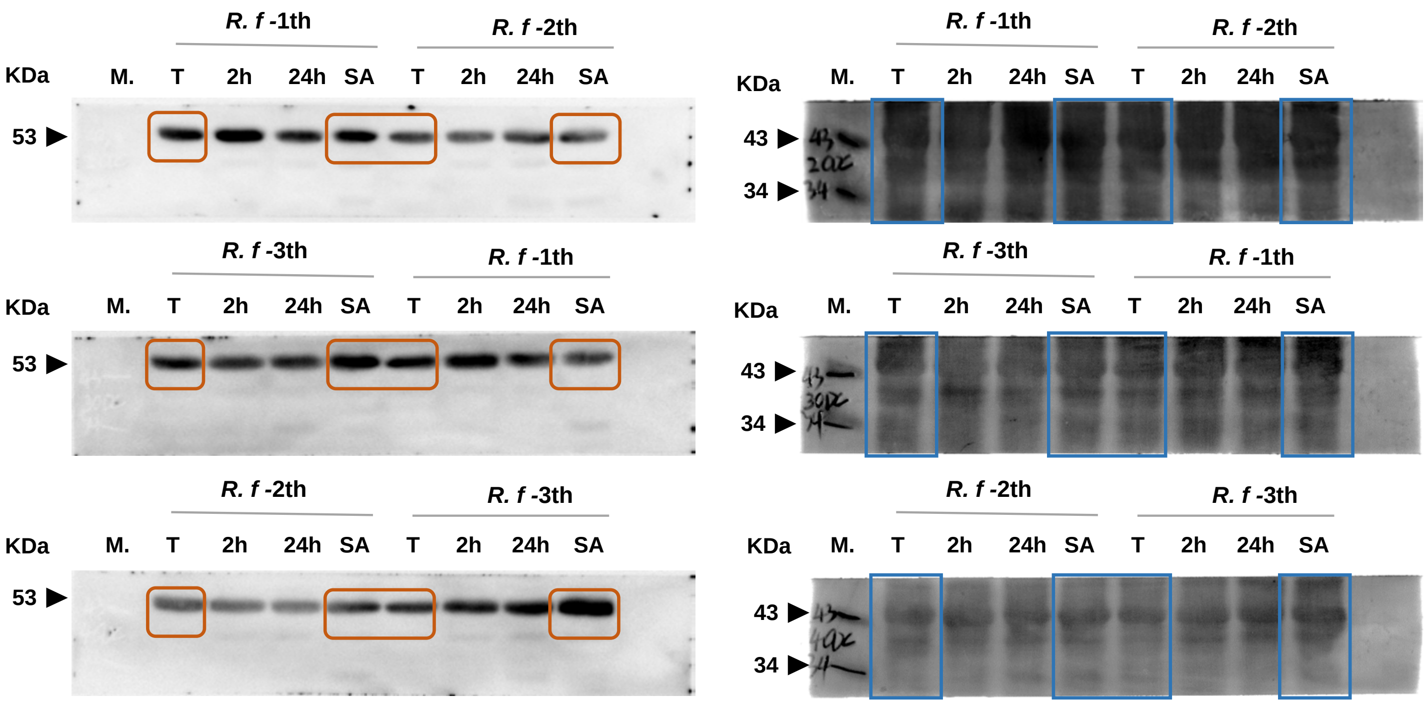

*Different numbers represent different bat individuals.
*M: marker, T: torpor, 2h: 2 hours after arousal, 24h: 24 hours after arousal; SA: summer active state

AGMAT

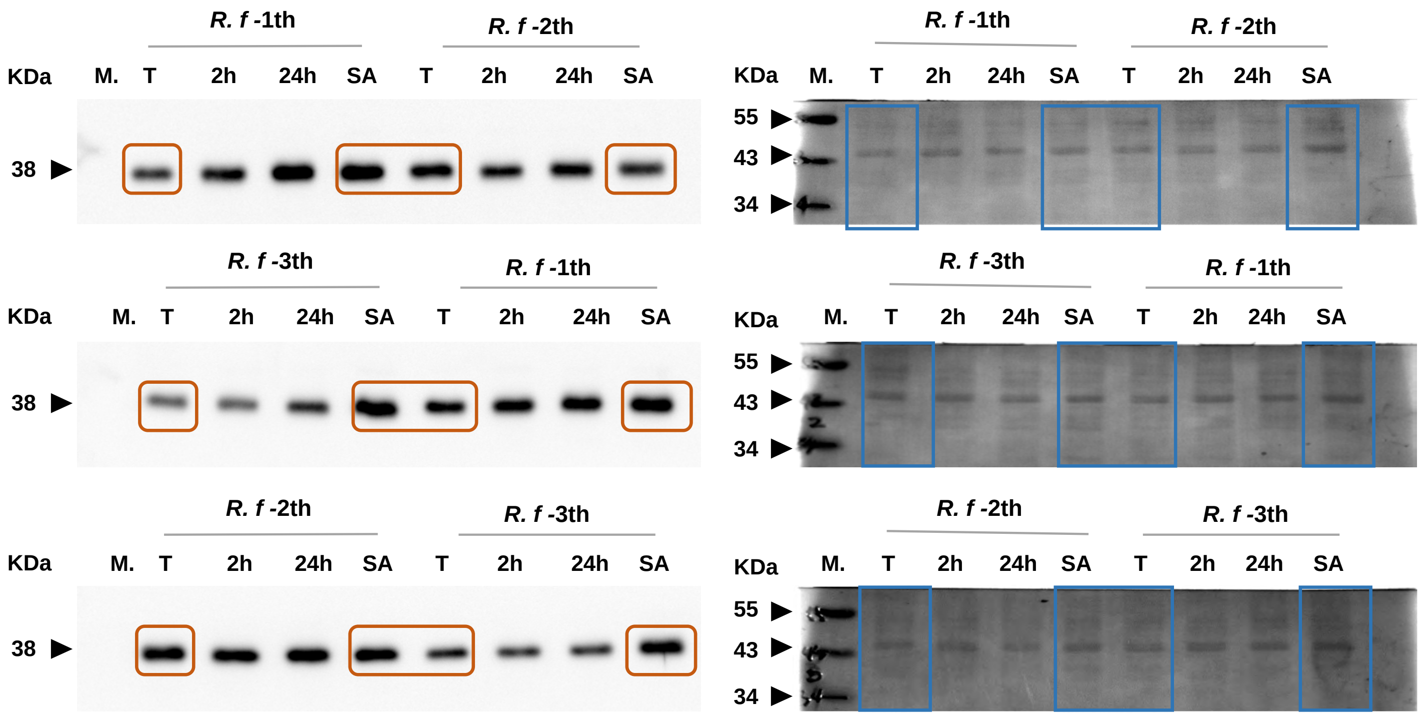

*Different numbers represent different bat individuals.
*M: marker, T: torpor, 2h: 2 hours after arousal, 24h: 24 hours after arousal; SA: summer active state

AGMAT

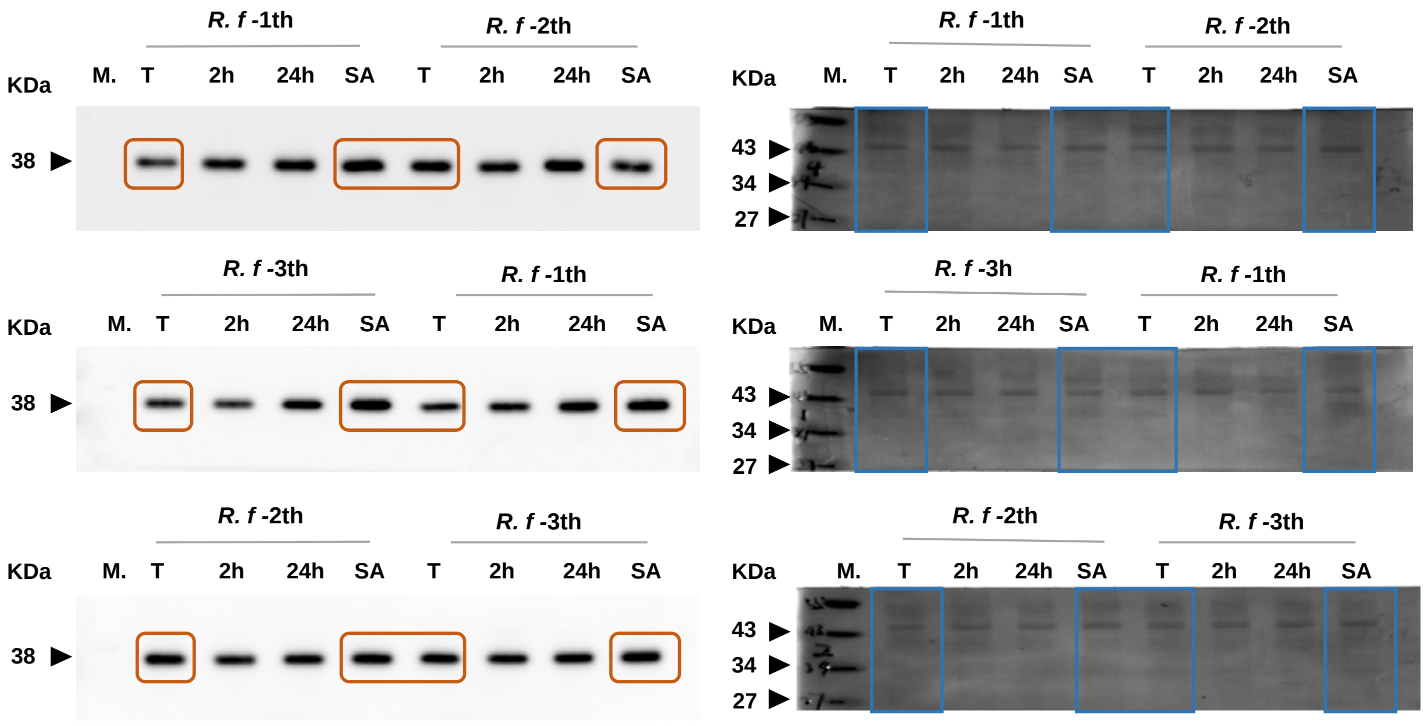

*Different numbers represent different bat individuals.
*M: marker, T: torpor, 2h: 2 hours after arousal, 24h: 24 hours after arousal; SA: summer active state

NAGS

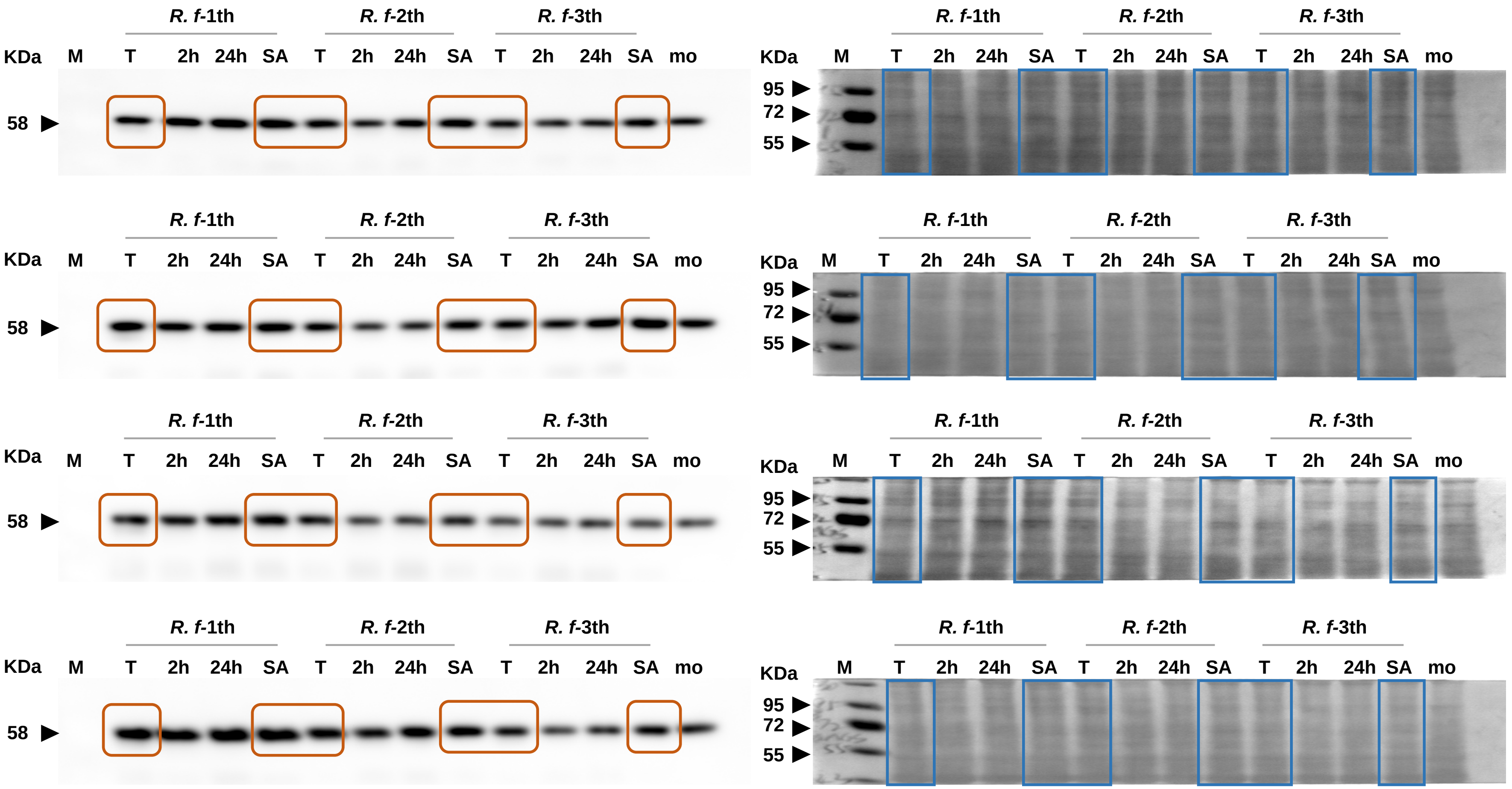

*Different numbers represent different bat individuals.
*M: marker, T: torpor, 2h: 2 hours after arousal, 24h: 24 hours after arousal; SA: summer active state
*mo: samples from mouse

NAGS

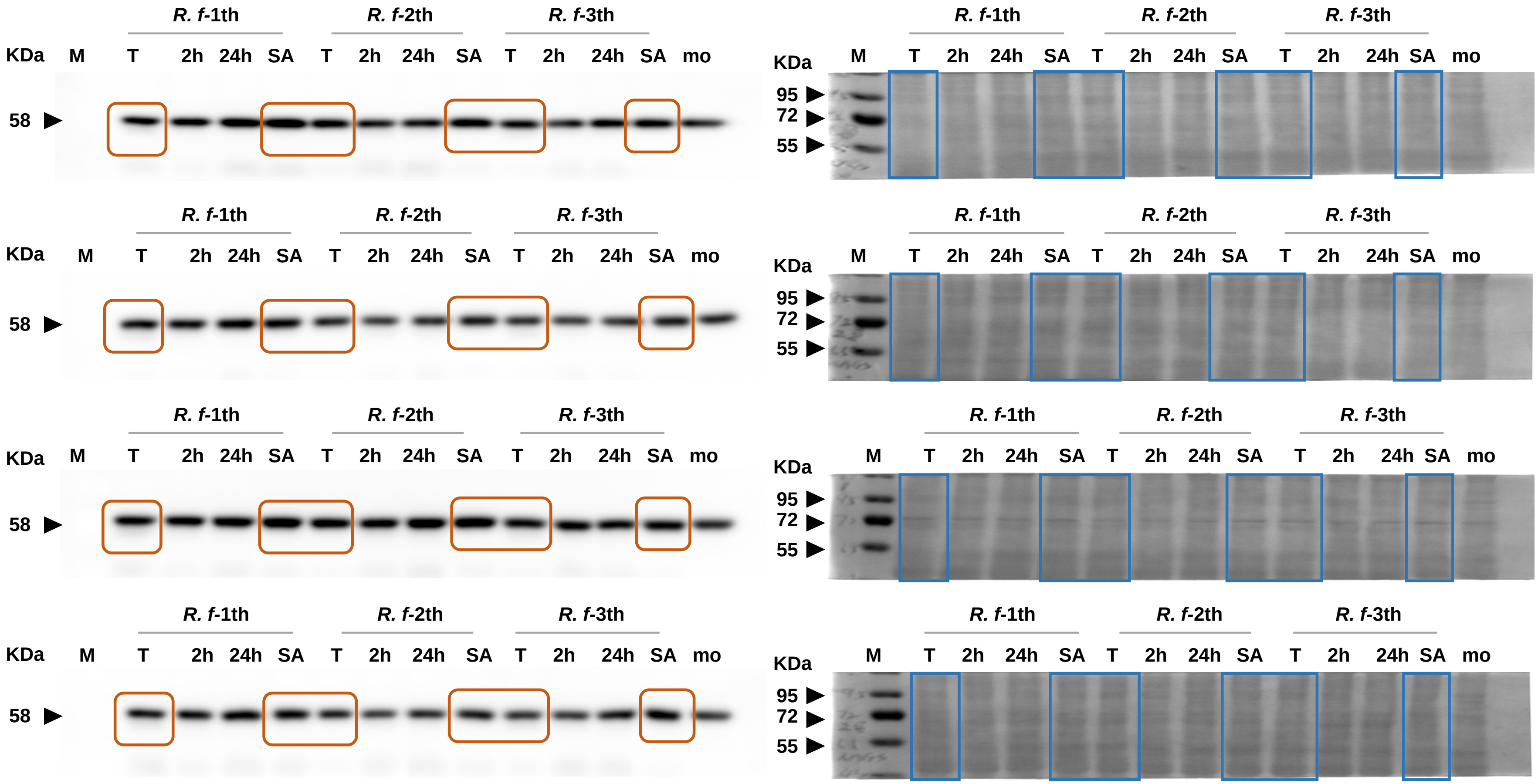

*Different numbers represent different bat individuals.
*M: marker, T: torpor, 2h: 2 hours after arousal, 24h: 24 hours after arousal; SA: summer active state
*mo: samples from mouse

SIRT5

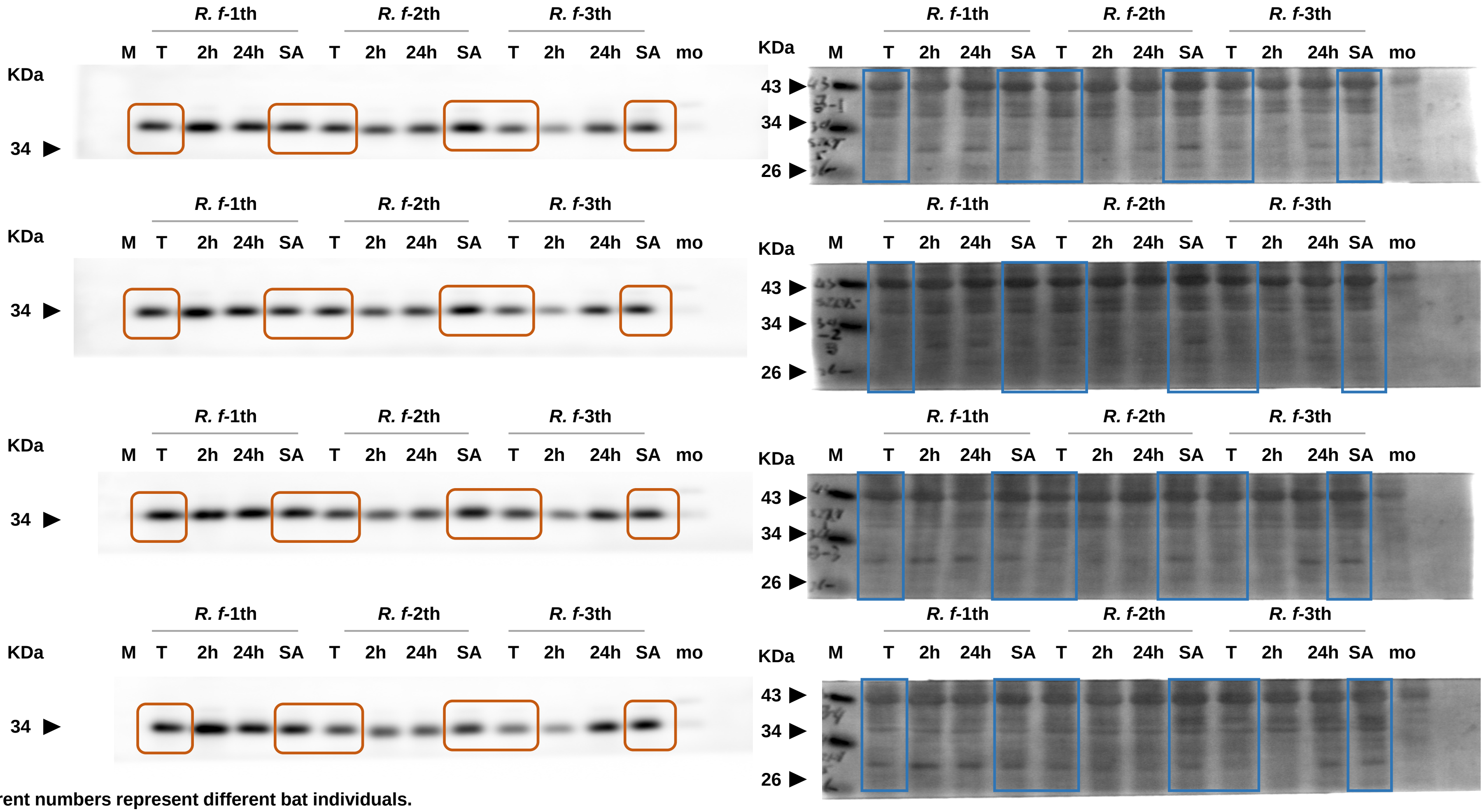

*Different numbers represent different bat individuals.
*M: marker, T: torpor, 2h: 2 hours after arousal, 24h: 24 hours after arousal; SA: summer active state
*mo: samples from mouse

SIRT5

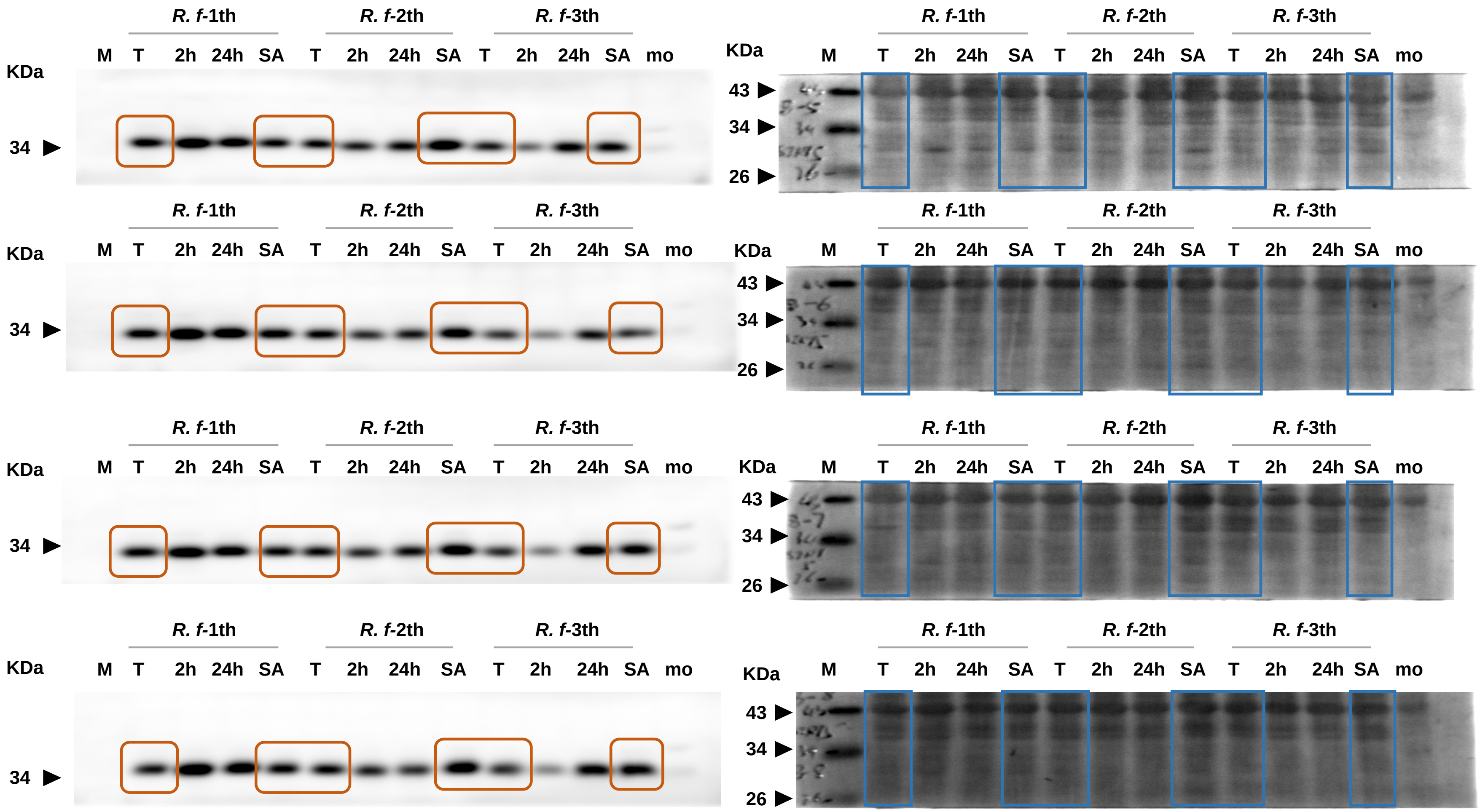

*Different numbers represent different bat individuals.
*M: marker, T: torpor, 2h: 2 hours after arousal, 24h: 24 hours after arousal; SA: summer active state
*mo: samples from mouse
