# Supplementary material for: Interaction of carbamoyl-phosphate synthase 1 with agmatinase in the liver of torpid bats
Source: Curr Zool. 2026 Jan 6;72(5):703–13. doi: 10.1093/cz/zoag002 (PMC13690593; doi:10.1093/cz/zoag002)
Supplement: Supplementary file 1 [file zoag002_supplementary_data.zip › Supplementary Information 2.pdf]

Co-localization experiments

3 individuals used for each species*: M. ricketti, R. ferrumequinum*, and *Mus musculus*

Myotis ricketti — Torpor

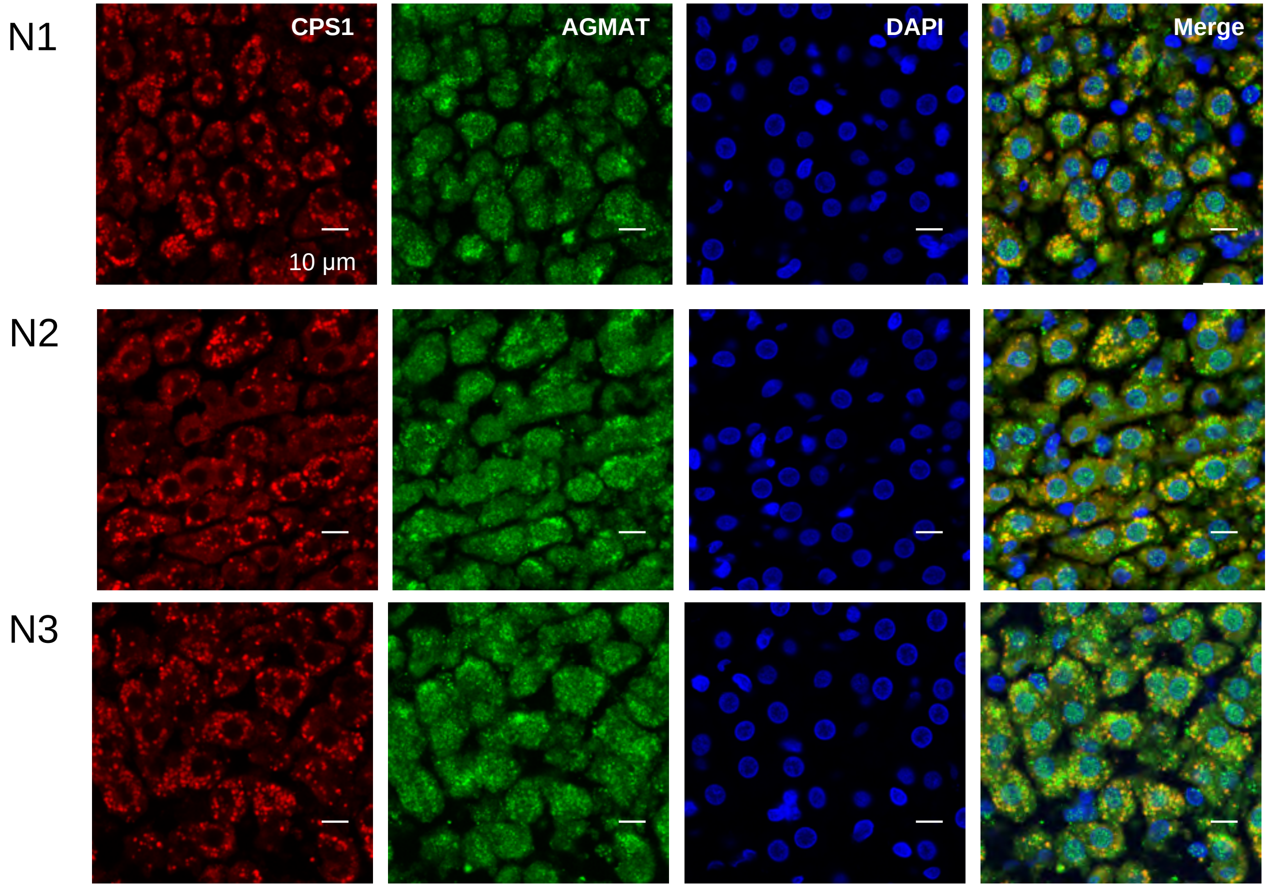

	PCC	M1	M2
**1**	0.654	0.477	0.914
**2**	0.654	0.456	0.907
**3**	0.622	0.39	0.938

N: Bat individuals
Whole image was used in calculation of PCC, M1, or M2

Myotis ricketti — summer active

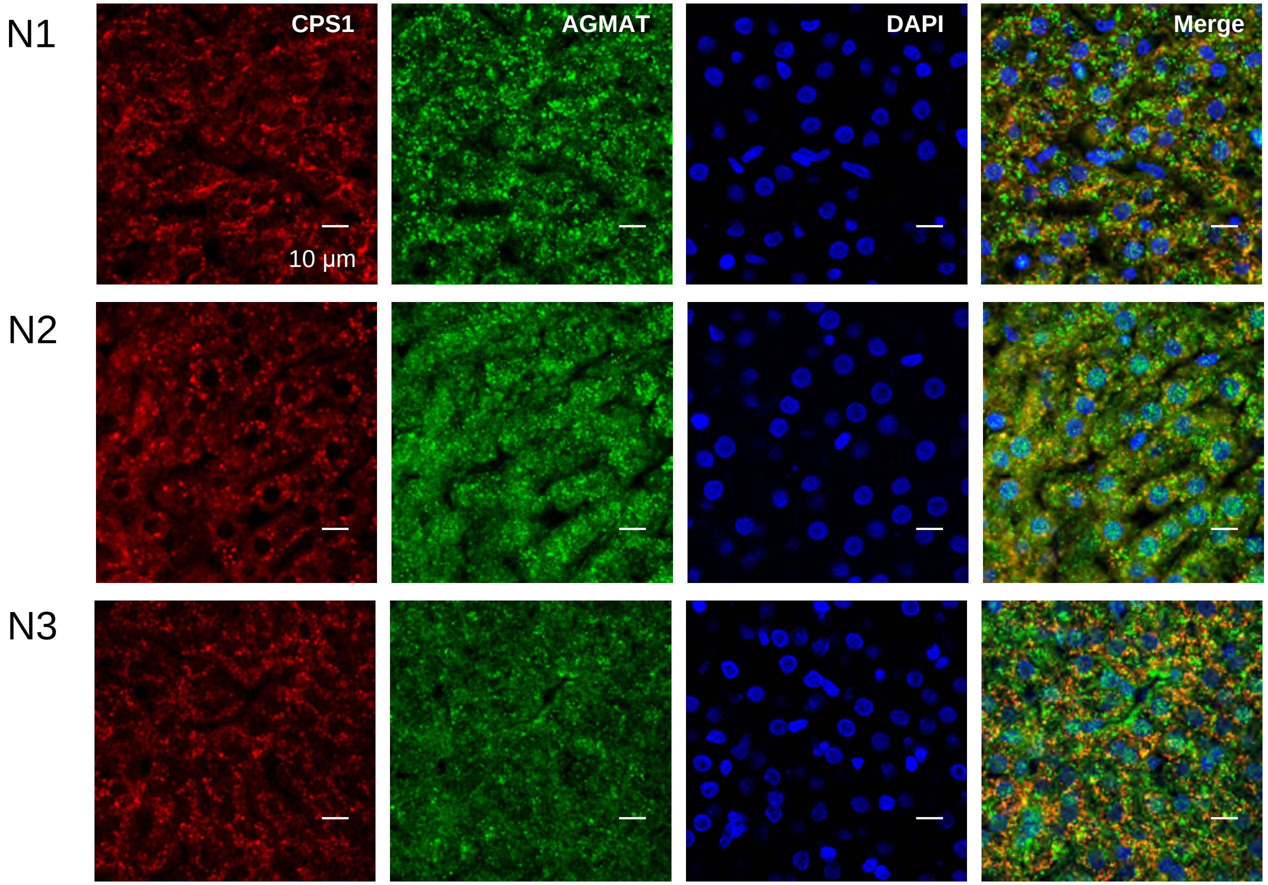

	PCC	M1	M2
**1**	0.414	0.51	0.585
**2**	0.399	0.505	0.738
**3**	0.353	0.335	0.706

N: Bat individuals
Whole image was used in calculation of PCC, M1, or M2

Rhinolophus ferrumequinum — Torpor

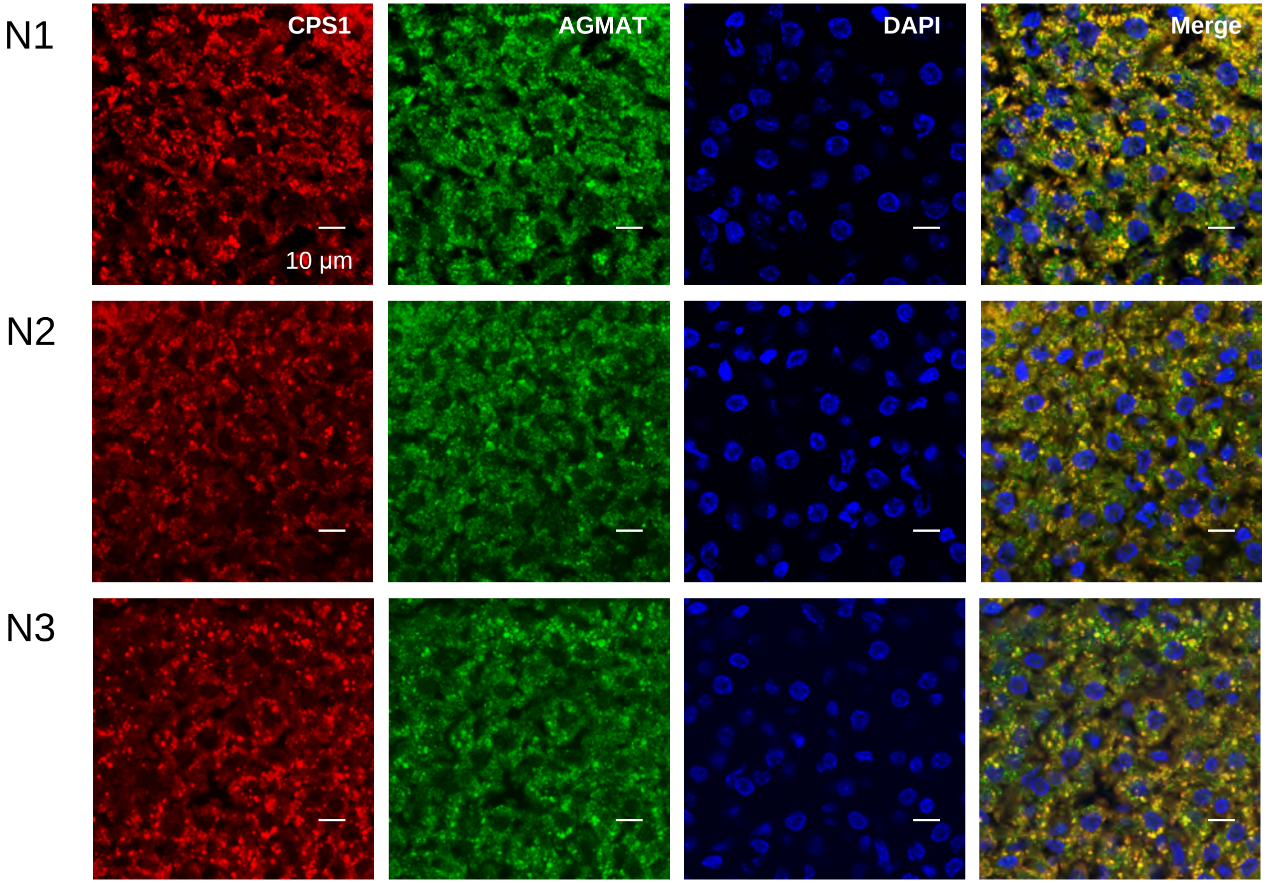

	PCC	M1	M2
**1**	0.805	0.577	0.942
**2**	0.73	0.516	0.922
**3**	0.767	0.499	0.929

N: Bat individuals
Whole image was used in calculation of PCC, M1, or M2

Rhinolophus ferrumequinum — summer active

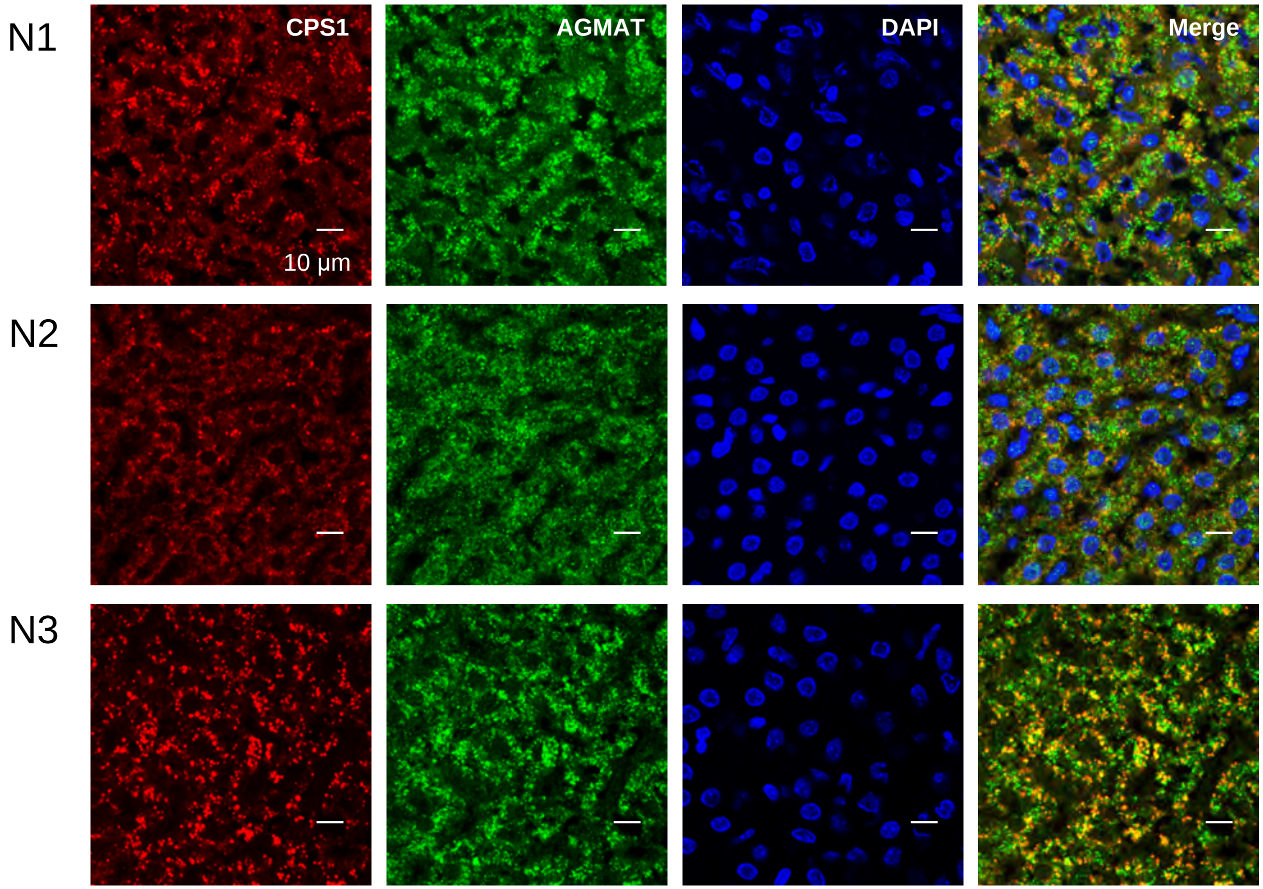

	PCC	M1	M2
**1**	0.493	0.516	0.707
**2**	0.501	0.561	0.744
**3**	0.619	0.421	0.816

N: Bat individuals
Whole image was used in calculation of PCC, M1, or M2

Mus musculus

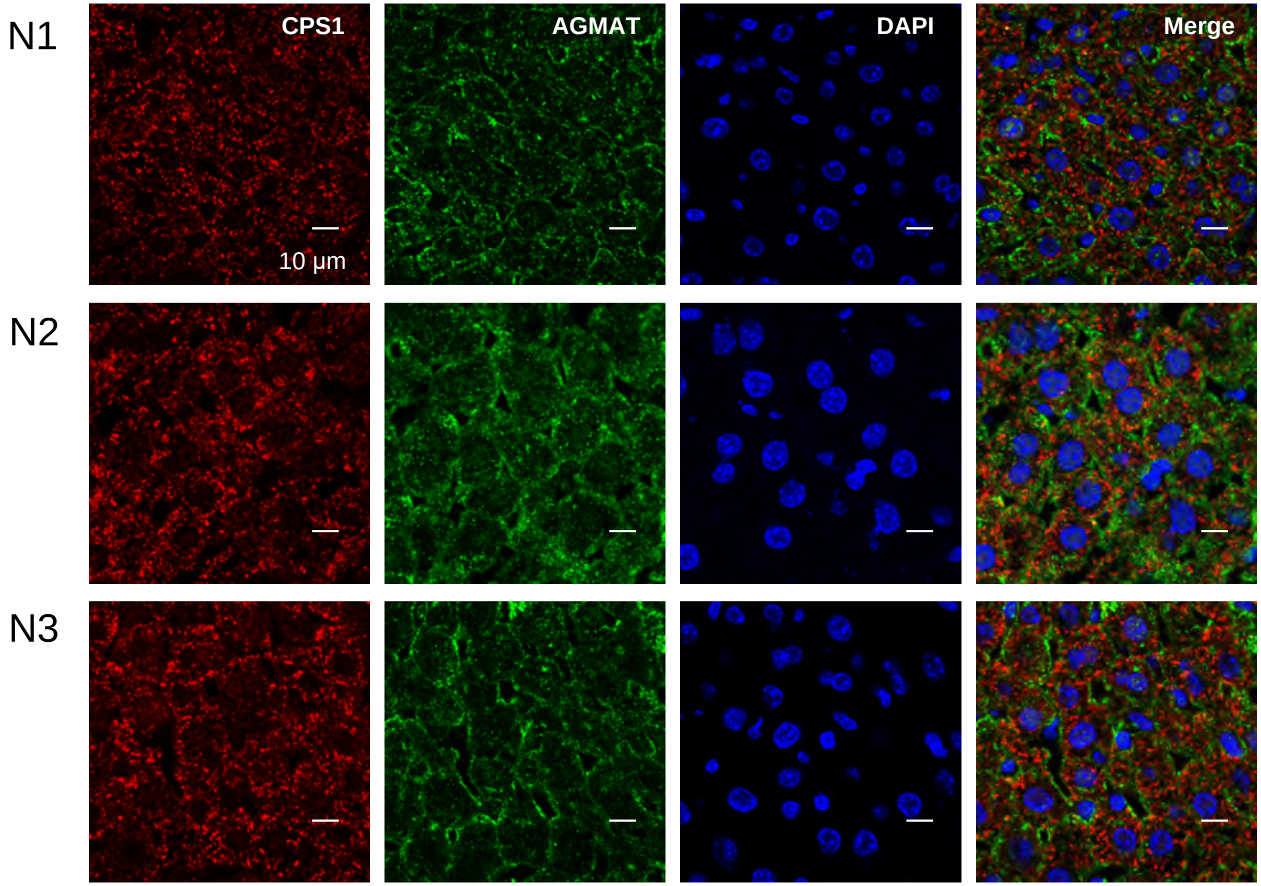

	PCC	M1	M2
**1-1**	0.134	0.27	0.363
**2-1**	0.164	0.305	0.264
**3-1**	0.178	0.268	0.398

N: mouse individuals
Whole image was used in calculation of PCC, M1, or M2
*Mice were used as a control species.
