# Supplementary material for: Interaction of carbamoyl-phosphate synthase 1 with agmatinase in the liver of torpid bats
Source: Curr Zool. 2026 Jan 6;72(5):703–13. doi: 10.1093/cz/zoag002 (PMC13690593; doi:10.1093/cz/zoag002)

2021

# *Myotis ricketti*

## Torpor – Individual 1

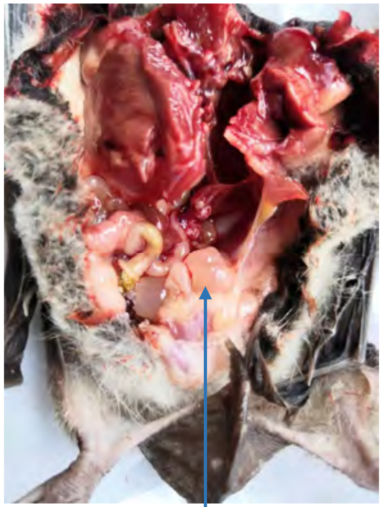

**Bladder**

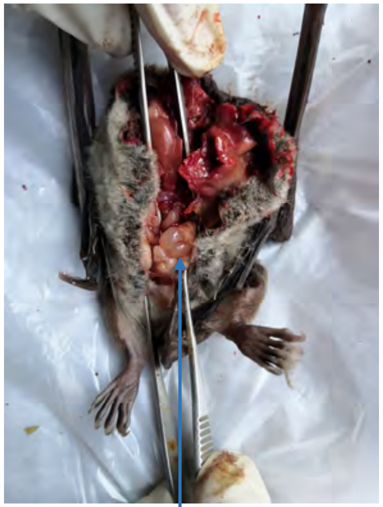

**Bladder**

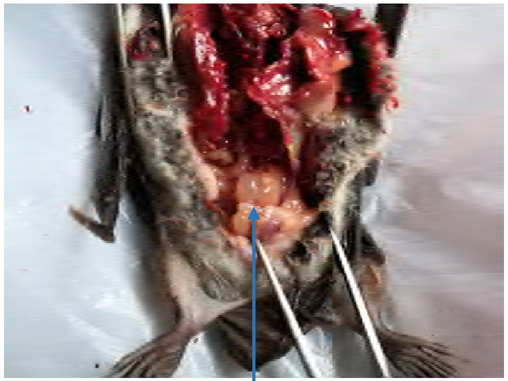

**Bladder**

# *Myotis ricketti*

## Torpor – Individual 2

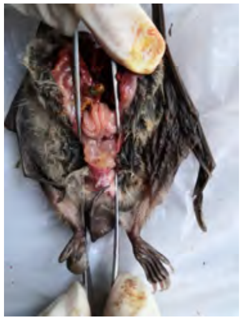
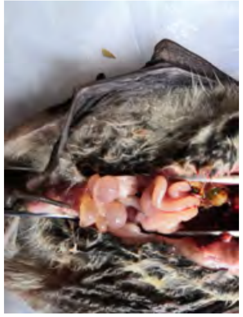
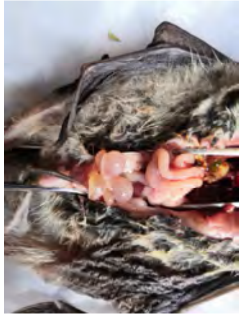
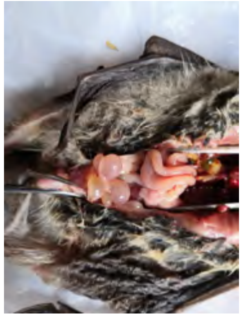
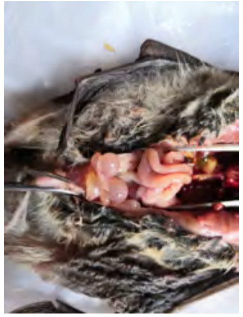

## Torpor – Individual 3

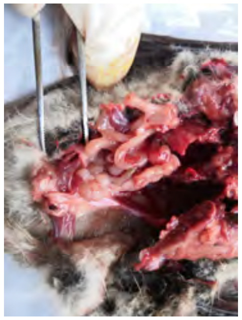
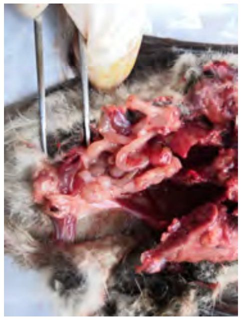
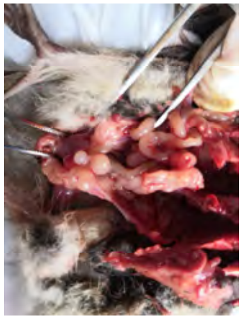

2021

# *Myotis ricketti*

## 2 h after arousal – Individual 1

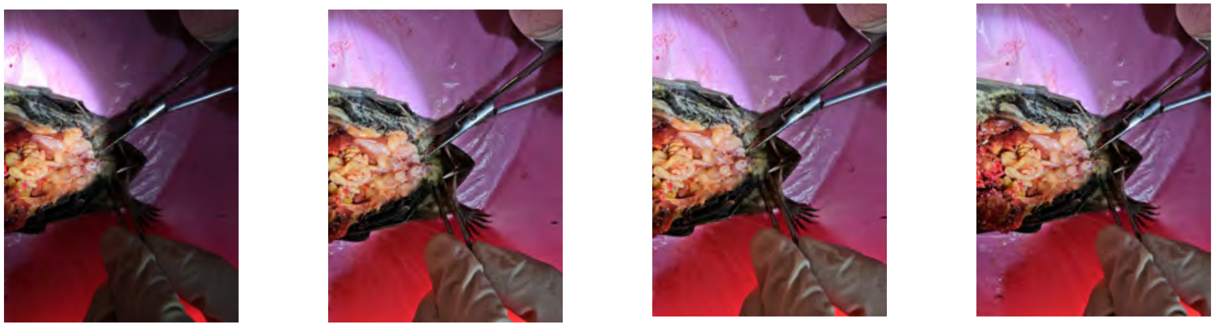

## 2 h after arousal – Individual 2

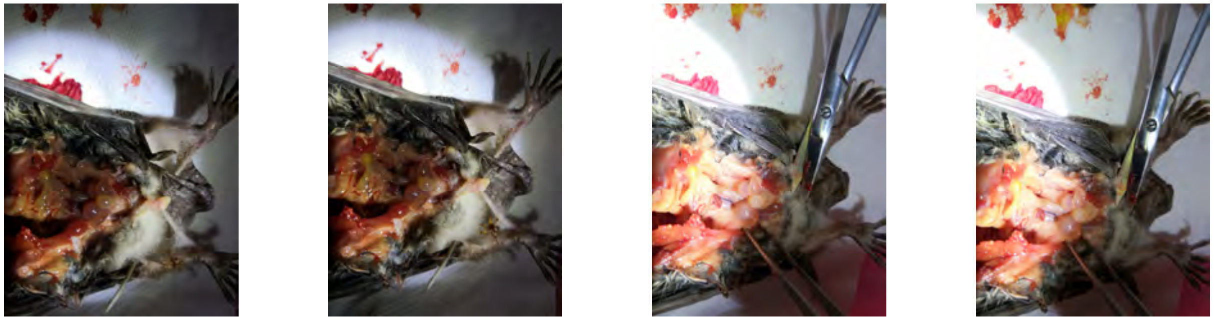

2021

# *Myotis ricketti*

## 2 h after arousal – Individual 3

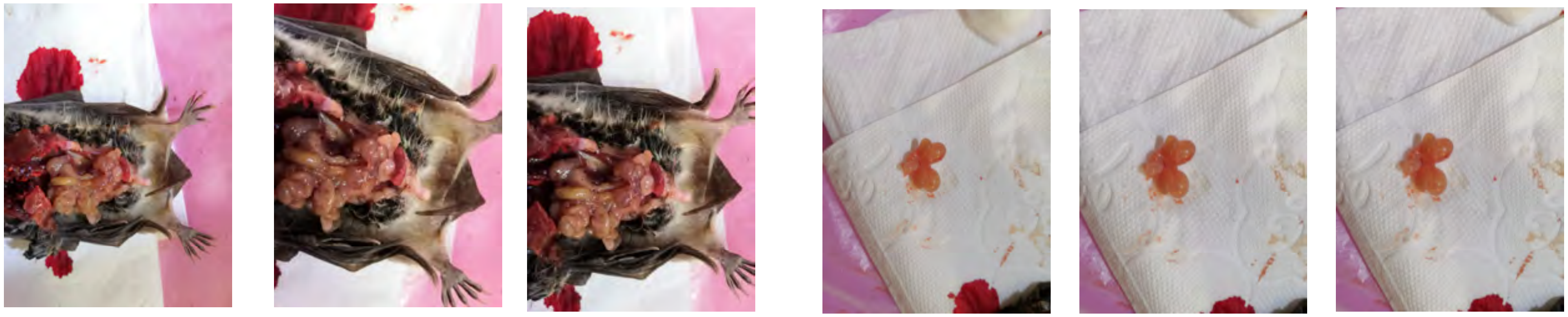

# *Myotis ricketti*

## 24 h after arousal – Individual 1

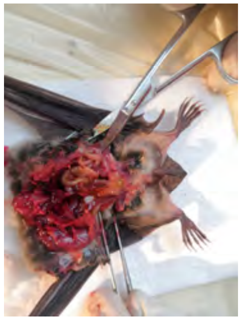
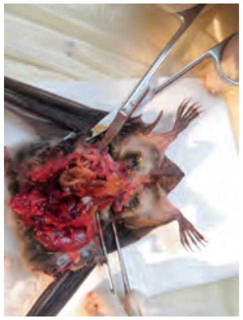
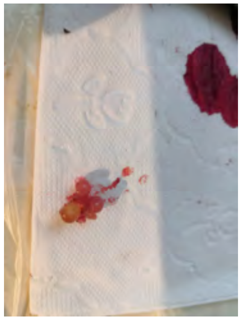
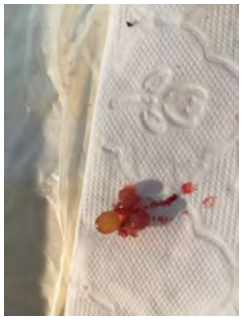

## 24 h after arousal – Individual 2

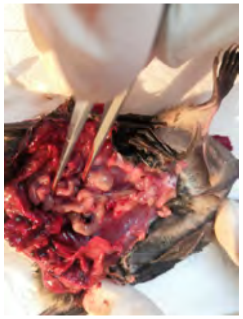
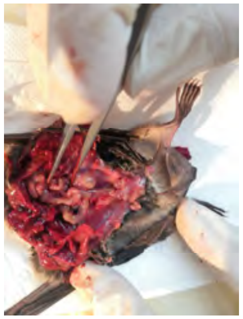
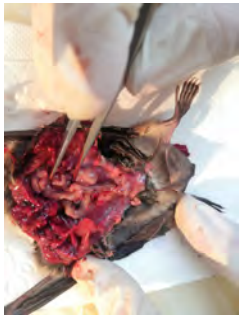
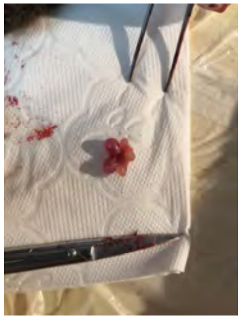
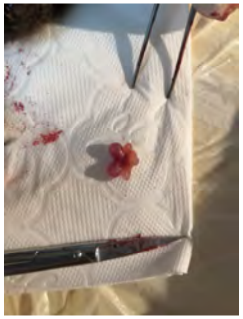
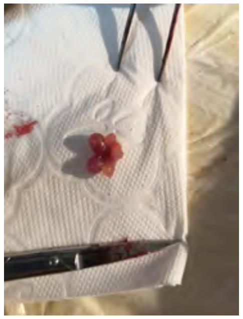

# *Myotis ricketti*

## 24 h after arousal – Individual 3

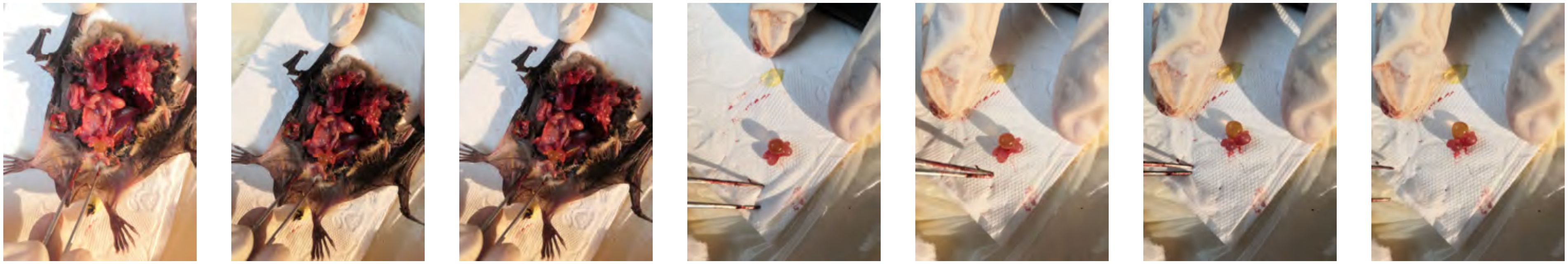

## 24 h after arousal – Individual 4

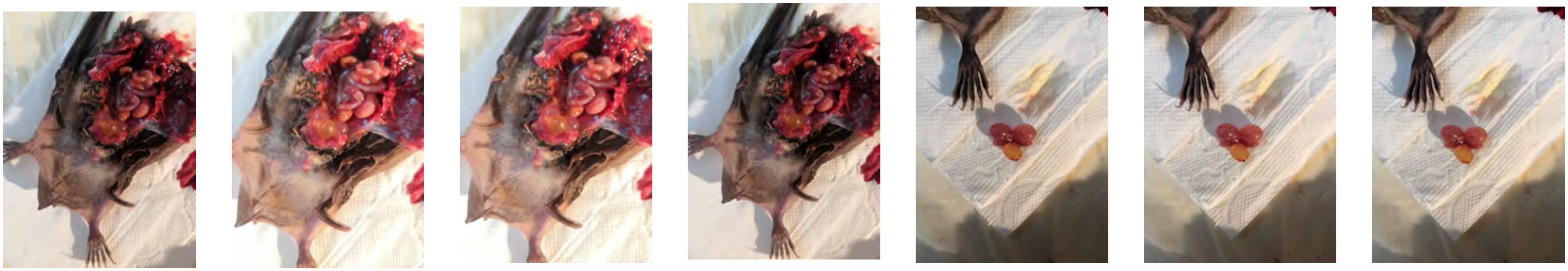

## 24 h after arousal – Individual 5

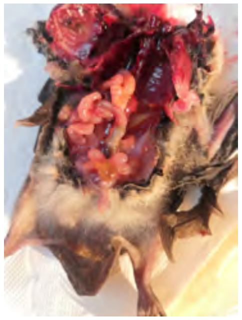
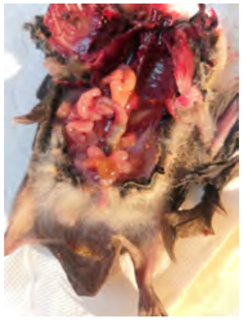
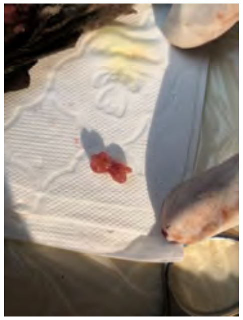
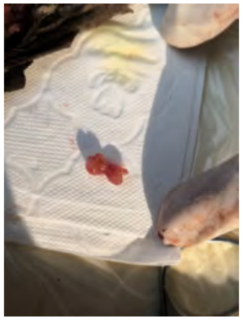

## 24 h after arousal – Individual 6

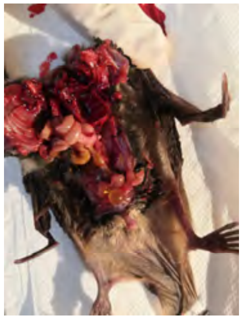
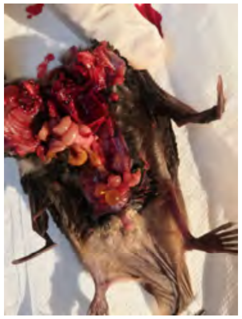
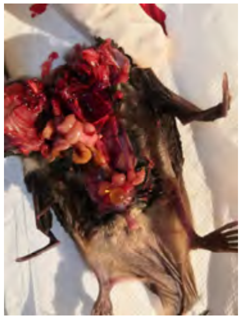
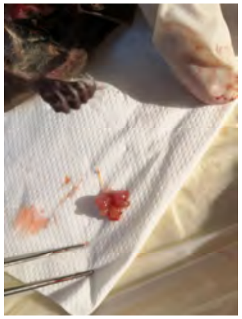
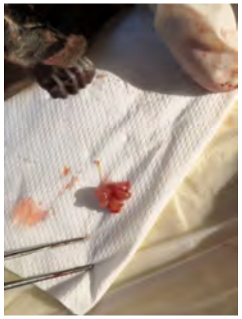
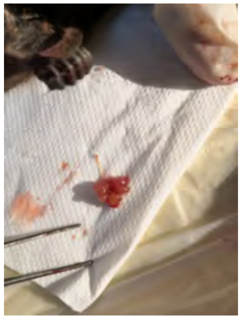

2021

# *Myotis ricketti*

## Summer active – Individual 1

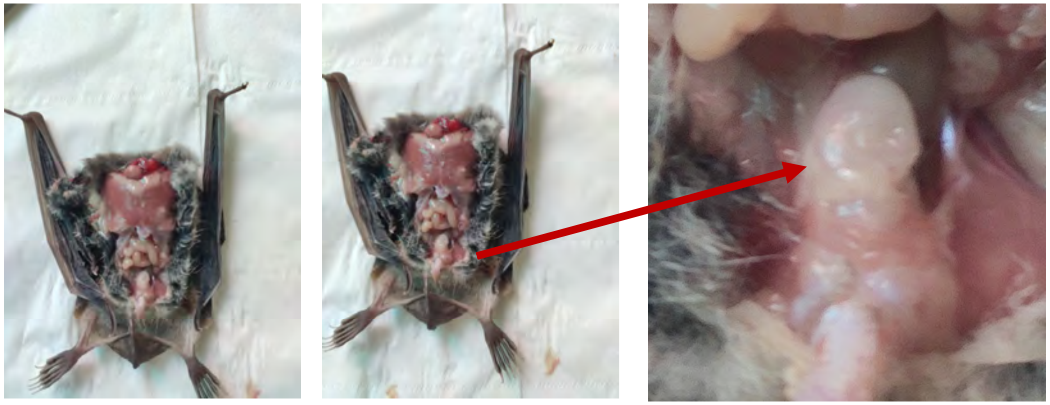

## Summer active – Individual 2

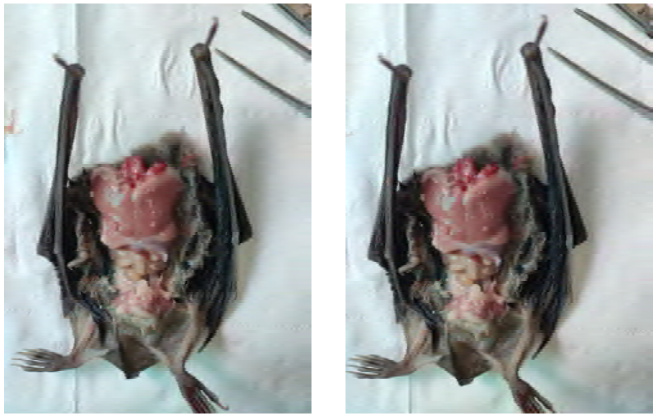

## Summer active – Individual 3

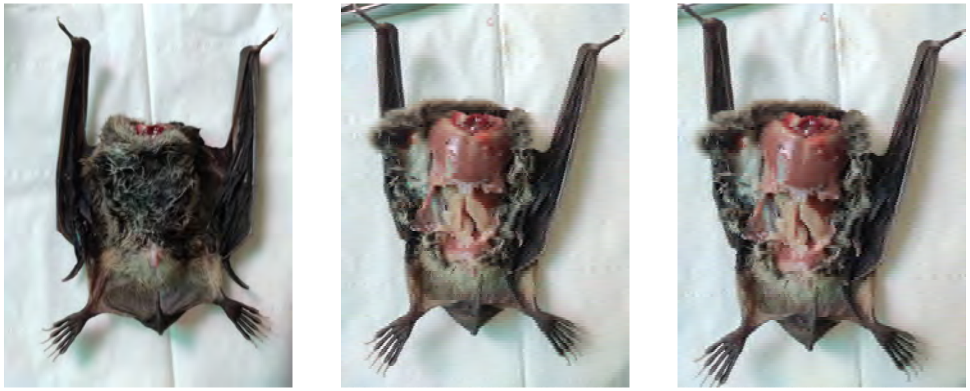

## Summer active – Individual 4

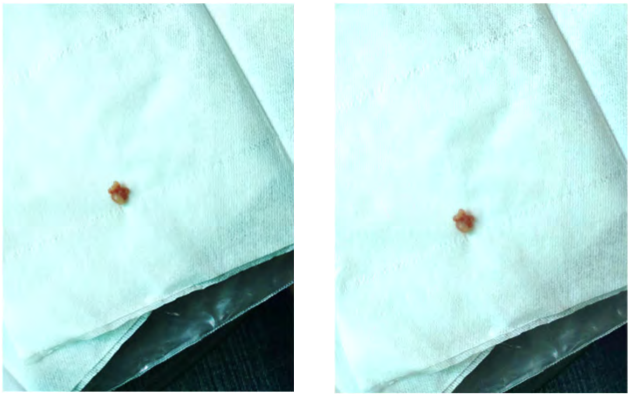

# *Myotis ricketti*

## Torpor – Individuals

1

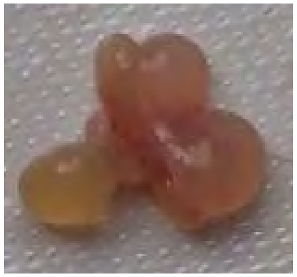

2

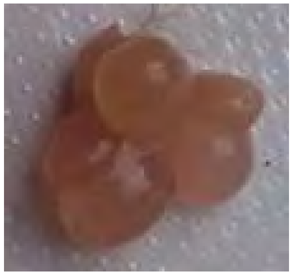

3

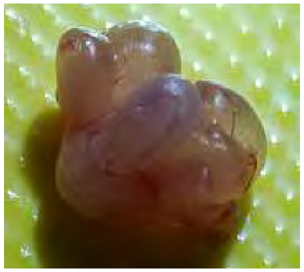

4

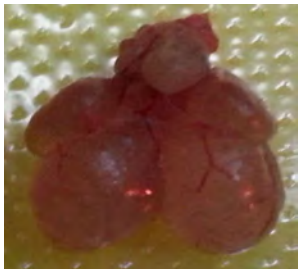

2021

# *Rhinolophus ferrumequinum*

**Torpor – Individual 1**

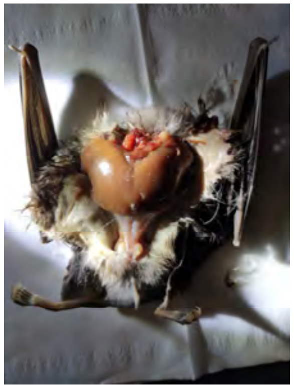

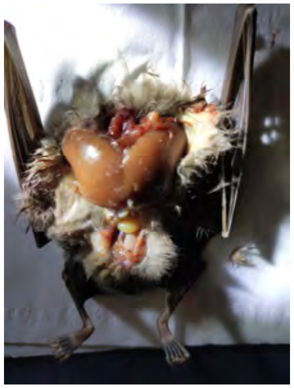

**Torpor – Individual 2**

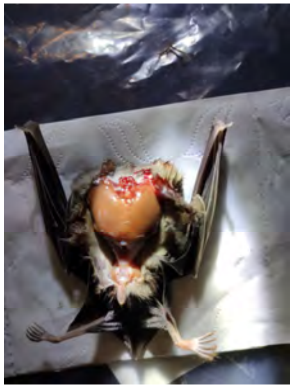

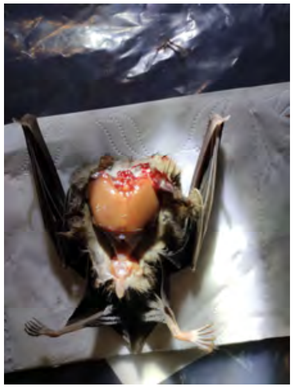

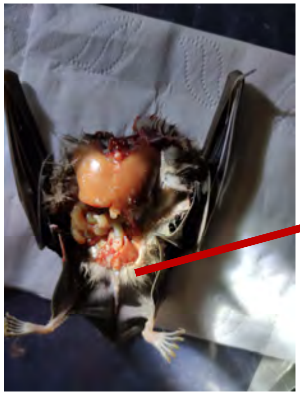

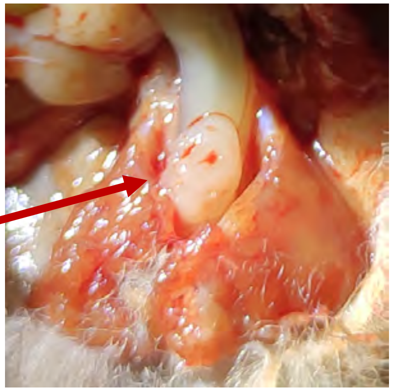

2021

# *Rhinolophus ferrumequinum*

## 2 h – Individual 1

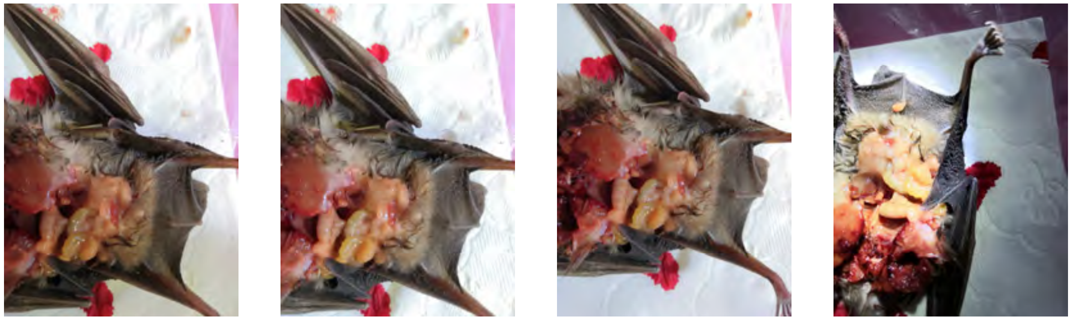

## 2 h – Individual 2

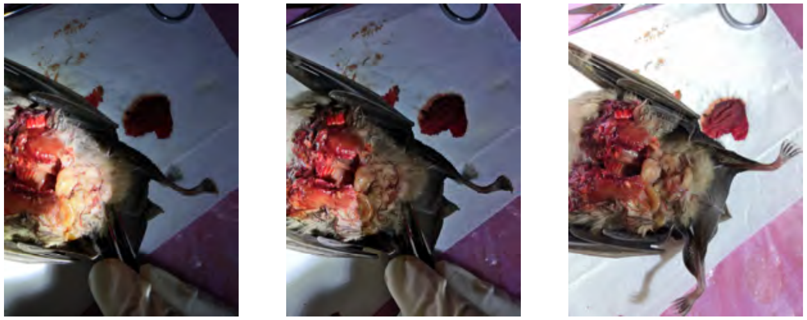

## 2 h – Individual 3

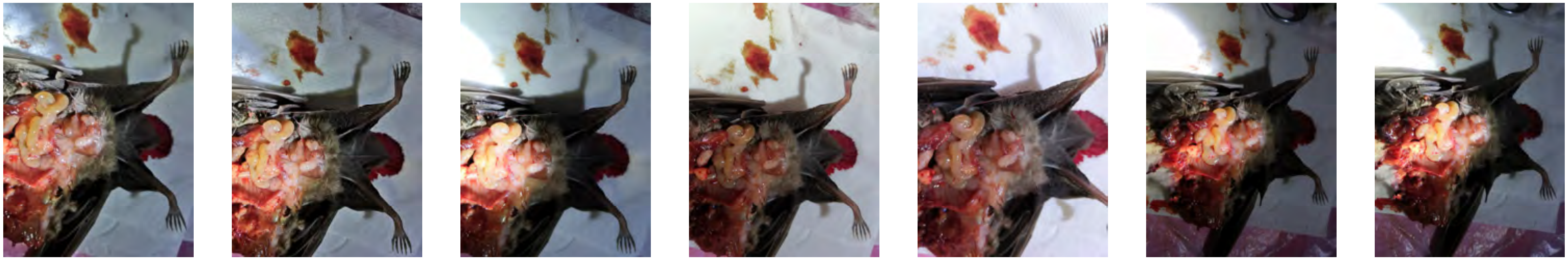

# *Rhinolophus ferrumequinum*

**24 h – Individual 1**

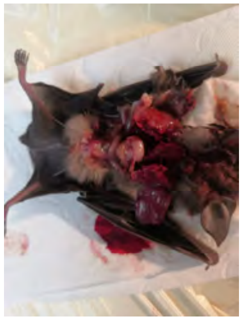

**24 h – Individual 2**

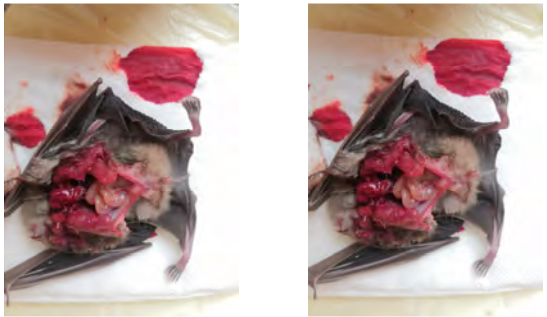

**Summer active – Individual 1**

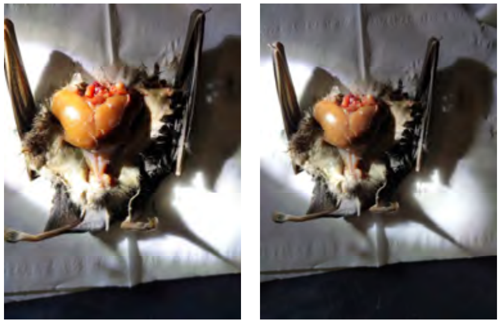

**Summer active – Individual 2**

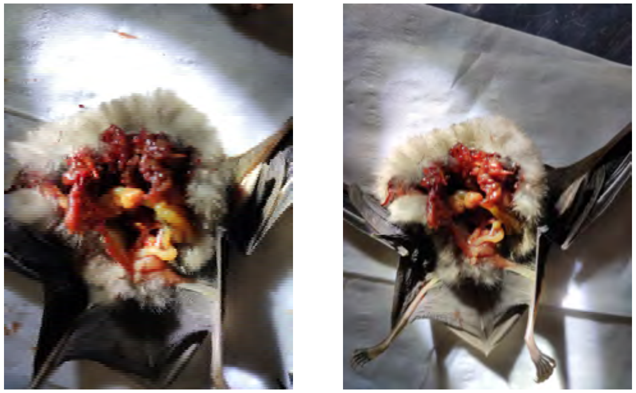

**Summer active – Individual 3**

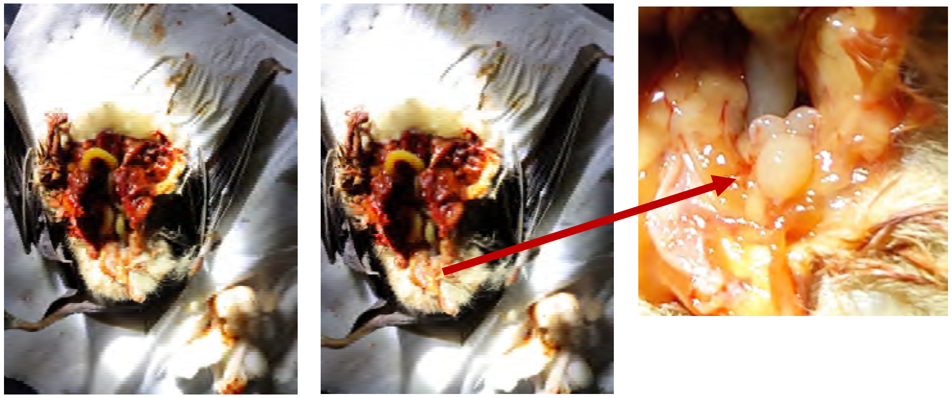

## *Mus musculus*

### Mouse – Individual 1

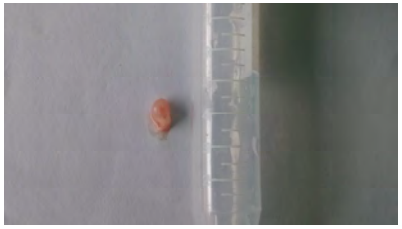

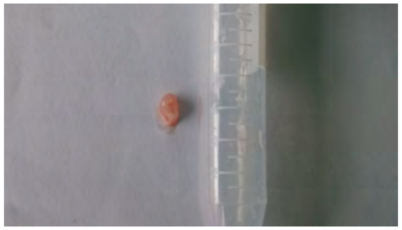

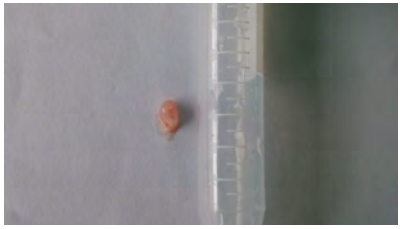

### Mouse – Individual 2

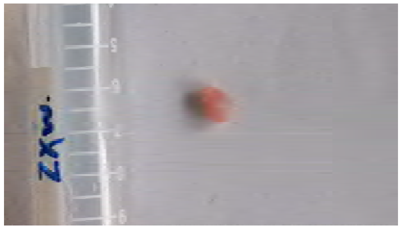

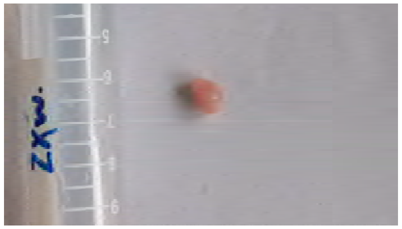

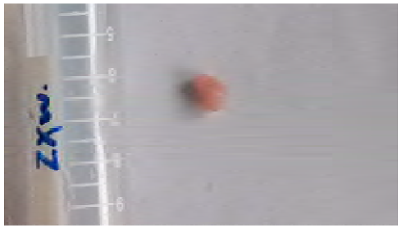

### Mouse – Individual 3

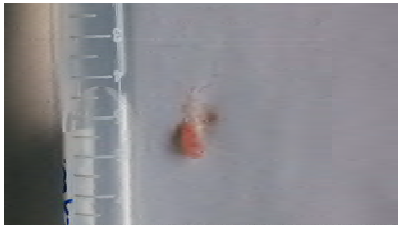

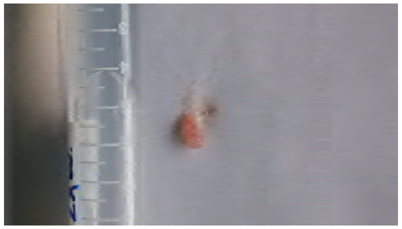

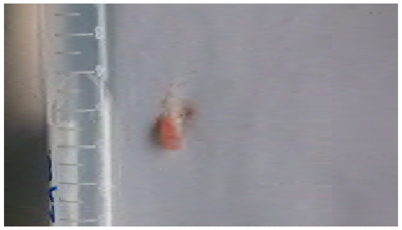

# *Mus musculus* (*db/db* mice)

## Mouse – Individual 1

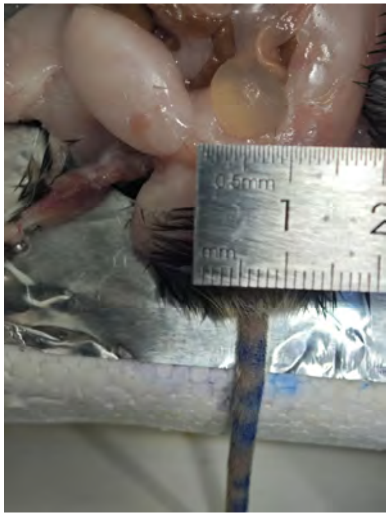


## Mouse – Individual 2

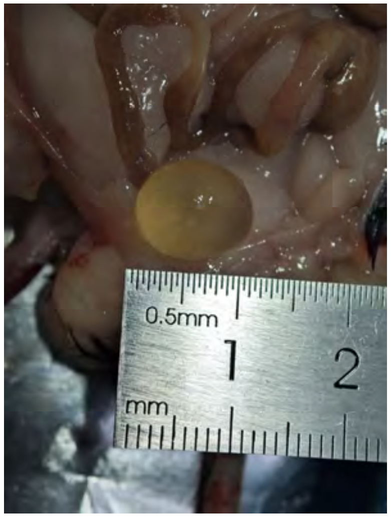


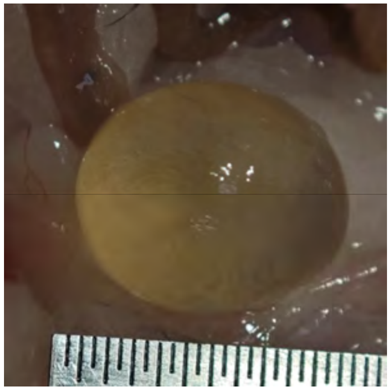

## Mouse – Individual 3

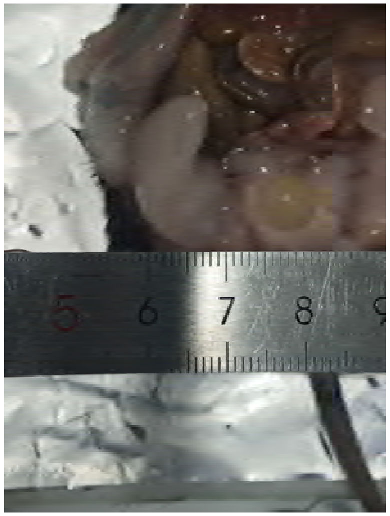

Supplement: Supplementary file 1 [file zoag002_supplementary_data.zip › Supplementary Information 3.pdf]
