# Supplementary material for: Interaction of carbamoyl-phosphate synthase 1 with agmatinase in the liver of torpid bats
Source: Curr Zool. 2026 Jan 6;72(5):703–13. doi: 10.1093/cz/zoag002 (PMC13690593; doi:10.1093/cz/zoag002)
Supplement: Supplementary file 1 [file zoag002_supplementary_data.zip › Table S1. Basic information of bats used in this study.docx]

**Table S1 Basic information of bats used in this study**

| **Species** | **States** | **Rectal temperature (^o^C)** | **Body mass (g)** |
| --- | --- | --- | --- |
| ***Myotis ricketti*** | Torpor | 8-13 | 15-17 |
|  | Summer active | 36-37 | 16-22 |
| ***Rhinolophus ferrumequinum*** | Torpor | 8-13 | 19-22 |
|  | Summer active | 36-37 | 16-21 |
